## [Editor Report]

The authors identified and characterized an m6A reader protein Pho92 in *Saccharomyces cerevisiae*, providing several lines of evidence suggesting that it functions in RNA decay and translation, using a combination of molecular biological and computational approaches. The work is of interest to molecular biologists who are interested in understanding how mRNA modifications affect cell fate decisions.

---

## [Decision Letter]

**Decision letter after peer review:**

[Editors’ note: the authors submitted for reconsideration following the decision after peer review. What follows is the decision letter after the first round of review.]

Thank you for submitting the paper "m6A reader Pho92 is recruited co-transcriptionally and couples translation efficacy to mRNA decay to promote meiotic fitness in yeast" for consideration by *eLife*. Your article has been reviewed by 3 peer reviewers, and the evaluation has been overseen by a Reviewing Editor and a Senior Editor. The following individual involved in the review of your submission has agreed to reveal their identity: Luke E Berchowitz (Reviewer #1).

Comments to the Authors:

We are sorry to say that, after consultation with the reviewers, we have decided to decline the paper in its current form. Nevertheless, we encourage you to resubmit when you have thoroughly addressed the reviewers' comments (see below).

All three reviewers had positive things to say about parts of the manuscript. However, all three also expressed significant concerns about other parts of the paper. The reviewers agreed that addressing these concerns would make the work publishable in *eLife*. Any revised manuscript will require a re-review.

*Reviewer #1:*

m6A is a pervasive and dynamic internal modification to polyadenylated RNA that is important for numerous physiological and pathological biological processes. However, the function of the m6A modification is often context-dependent and challenging to pin down. In the budding yeast *Saccharomyces cerevisiae*, m6A is exclusive to gametogenesis where it plays a major role in meiotic progression. Because deletion of the lone RNA methyltransferase (IME4) exerts a clear phenotype in an inessential process (i.e. meiosis), *S. cerevisiae* is an ideal organism to investigate m6A biology. Varier et al. set out to identify proteins that bind to m6A ("readers") in *S. cerevisiae* meiosis and then to understand how the binding of these readers affects the fate of m6A-modified mRNA.

The authors use an m6A bait/mass spectrometry-based approach to determine that Pho92 binds to m6A-modified transcripts as a potential reader. The interaction between Pho92 and m6A-modified RNA is extensively validated. All the Pho92 CLIP experiments include a powerful control (ime4 deletion). This allows the authors to determine which Pho92-RNA interactions require m6A modification. Because of this control and the observation that Ime4-dependent Pho92 RNA binding sites are highly enriched at m6A (based on miCLIP data), the idea that Pho92 binds, in part, to m6A-modified RNA is properly supported. The next question is key – what is the function of the Pho92-m6A interaction? The authors used a clever experiment to test the idea that Pho92 accelerates the decay of m6A-marked transcripts. They blocked transcription and then measured the decay rate of m6A compared to adenosine (unmodified is my understanding). Strikingly, they found that m6A decays more rapidly than unmodified A in a manner that requires Pho92. In my opinion, this is the key result of the paper because it gives insight into both the function of m6A itself in meiosis and the function of the Pho92-m6A interaction. While these population-based measurements do not explain the fate of every m6A-marked transcript, the trend of increased decay rate is important. In my opinion, the idea that Pho92-mediated decay of m6A-marked RNA requires translation is partially supported by subtle effects on protein/RNA ratios. While this idea may turn out to be correct, the data shown do not constitute a slam dunk.

My main reservation about the paper (in this form) is based on the circumstance that pho92 mutants exhibit defective respiratory growth. Because respiration is critical for meiosis in yeast, I wonder whether and to what degree the effects the authors observe in the pho92 deletion background (m6A decay, meiotic progression, gamete viability, etc.) are due to problems in respiration rather than m6A reader function. I know I'm not supposed to recommend experiments here, but epistasis analyses would be able to address this concern.

As I mentioned earlier, this solid paper constitutes a timely and important contribution to understanding the function of m6A and reader proteins. In my opinion, RNA modification papers often get bogged down in lists and dynamics without providing convincing data to address the difficult question of the function of the modification. This paper provides substantial support to the idea that m6A drives the decay of specific transcripts in a manner that requires interaction with an RNA-binding protein. It will be exciting for future studies to determine why it is important for cells to accelerate the decay of these transcripts to promote meiotic progression.

I enjoyed this paper overall. While I think the ideas put forth in the paper are likely correct, my main concern is the degree to which the observations in the pho92 mutant are due to respiratory defects rather than reader function. I think repeating some of the key experiments in a separate mutant with a similar respiratory defect would be very useful.

Along the same lines, some epistasis experiments could strengthen the conclusions of the paper. First, as you mentioned, it would be more apt to compare meiotic progression in the pho92 mutant to the ime4 catalytic dead rather than the full deletion. I think it would be useful to analyze meiotic phenotypes in a pho92; ime4 catalytic dead double mutant. This would help answer what proportion of the pho92 meiotic phenotype depends on m6A. If the ime4 cat-dead progression defect is epistatic to the pho92 defect (or vice versa), it would constitute strong support for the conclusions of the paper.

Similarly, the idea that Pho92-mediated decay of m6A transcripts requires CCR4-NOT would be strengthened by analysis of the pho92; not3 double mutant (Figure 5F, G). The key prediction is that decay of m6A should not be further reduced by pho92 deletion in a not3 mutant.

*Reviewer #2:*

Varier et al. identified Pho92 as an m6A reader protein using differentially labeling followed by mass spec in this manuscript. They revealed that Pho92 associates with the 3'end of meiotic mRNAs in an m6A-dependent and independent manner using CLIP-seq. They show that Pho92 interacts with Paf1C in the nucleolus while it promotes m6A-dependent mRNA decay via the CCR4-NOT complex. They suggested a model that m6A enhances protein synthesis and mRNA decay to facilitate meiosis. The function of m6A in *Saccharomyces cerevisiae* is much less known than in other species. Identifying a new reader protein and providing insights into its possible biological functions are innovative and significant to the field. The authors also combined a battery of cell biology, biochemical, and sequencing approaches to provide convincing conclusions that Pho92 is an m6A reader and its possible role in RNA decay.

• The authors need to revise m6A as N6-methyladenosine (m6A).

• The authors wrote "m6A modified (GGAmCU) or unmodified (GGACU)" which might be misleading. Am is likely interpreted as 2'O methylation, but I think the authors meant methylation in the base.

• Identifying Pho92 as an m6A reader is a success; it is a YTH protein homolog that makes the discovery very convincing together with all the sequencing and biochemical data. However, the authors claimed that Pho92 is likely the only m6A reader, which could be right or wrong. YTHDF and YTHDC proteins have been considered the "only" m6A readers in humans for a few years, but later more "m6A readers" were identified. Pho92 did not completely cover IME2 opens the possibility of additional m6A readers.

• Figure 3A, the authors could compare human METTL3/14 data with YTHDF proteins to compare with IME4 overlap with Pho92.

• Can the authors comment on why only 30% of m6A sites are consistent with published data sites?

• Can the authors provide the overlap percentage of m6A sites identified using miCLIP and ime4-dependent sites?

• The author claims that Paf1C contributes to the localization of Pho92 to the nucleus, and m6A modified transcripts facilitate the transition of their reader Pho92 into the cytoplasm. This conclusion is not supported, there are many other possibilities.

• Pho92 associates with nascent nuclear RNA in a Paf1C-dependent manner, however, what is the function of Pho92 interacting with nascent RNA? Do the authors hypothesize Pho92 works like YTHDC1, which facilitate m6A translocation and m6A-dependent splicing? These data on Paf1C and Pho92 are floating without conclusive function as it is currently presented.

• The impact of Pho92 on RNA decay is very convincing. However, the impact on translation is less compelling. The differences are tiny in protein level (Figure 6H), which was similarly seen in human YTHDF1 (promotes translation under stimuli)

• Overall, it is a great study and provided many lines of useful information. However, this paper is trying to say that Pho92 covers all known human m6A readers' functions. The authors might consider organizing and emphasizing the best data.

*Reviewer #3:*

In this manuscript, Varier et al. sought to characterize the protein(s) that mediate the function of the RNA modification m6A during meiosis in *Saccharomyces cerevisiae*. They identified Pho92 as likely the sole m6A reader in this organism and showed that it is important for the onset of meiosis, as previously found for the enzyme Ime4 that installs m6A. Next, they showed that Pho92 is likely recruited co-transcriptionally through direct binding to the transcription elongation complex Paf1C. Finally, they found that Pho92 promotes both the decay of m6A-modified transcripts as well as their translation. The decay appears to be contingent on the translation process. The amount of data presented in the paper is impressive and supports well the conclusions of the authors. One of the unclear parts concerns the connection between the Paf1C complex and Pho92, and the second is the indirect approach that is used to monitor mRNA decay.

1. By eye it is difficult to appreciate the re-localization of Pho92 in the cytoplasm after IME1 induction (Figure 4D). Furthermore, the data is not shown for the different mutant conditions (only the quantification is shown). This system is rather artificial as Pho92 is normally not expressed at 0h and 2h after induction. Could the authors instead check the localization of endogenous Pho92? By staining or fractionation experiment?

2. The decreased binding of Pho92 to chromatin upon leo1 depletion may not be related to their direct interaction. Another explanation could be that leo1 affects transcription, and therefore less m6A modified transcripts would be produced, which would decrease the binding of Pho92 to chromatin. To discard this possibility the authors should check the RNA level upon leo1 depletion, and also check whether the binding of Pho92 is independent of the nascent transcripts. I also note that the decreased binding is not supported by statistics.

3. It is unusual to quantify the level of m6A instead of the transcript itself to demonstrate a role in mRNA decay. If the effect is visible at the m6A level it should also be visible at the transcript level. I am a bit confused and not convinced with this indirect approach.

---

## [Author Response]

[Editors’ note: the authors resubmitted a revised version of the paper for consideration. What follows is the authors’ response to the first round of review.]

Reviewer #1:I enjoyed this paper overall. While I think the ideas put forth in the paper are likely correct, my main concern is the degree to which the observations in the pho92 mutant are due to respiratory defects rather than reader function. I think repeating some of the key experiments in a separate mutant with a similar respiratory defect would be very useful.

The reviewer points out the *pho92*∆ may have a respiratory defect. This was also indeed annotated as a phenotype for *pho92*∆ at yeastgenome.org database. Based on the reviewer’s suggestion, we have determined how the *pho92*∆ performs under respiratory growth conditions. Specifically, we have monitored growth of *pho92*∆ and wild type using different nonfermentable carbon sources, such as acetate or glycerol. We found no growth defect in pho92∆ cells when cells were spotted on YP + glycerol agar plates and have now included this data as a panel in Figure 3- Supplemental 2A. Also, the doubling time in YP + acetate was similar between WT and pho92∆ cells, which we have commented on in the main text. Our results strongly suggest that *pho92*∆ have no obvious respiratory defect.

Along the same lines, some epistasis experiments could strengthen the conclusions of the paper. First, as you mentioned, it would be more apt to compare meiotic progression in the pho92 mutant to the ime4 catalytic dead rather than the full deletion. I think it would be useful to analyze meiotic phenotypes in a pho92; ime4 catalytic dead double mutant. This would help answer what proportion of the pho92 meiotic phenotype depends on m6A. If the ime4 cat-dead progression defect is epistatic to the pho92 defect (or vice versa), it would constitute strong support for the conclusions of the paper.

We compared the delay in meiotic progression between *pho92*∆ and the *IME4* catalytically inactive mutant. We observed that the single and double mutants have a comparable delay in the onset of meiosis, thereby suggesting that the catalytic function of *IME4* and Pho92 function are epistatic. In addition, we generated a strain expressing Pho92 with a point mutation in its YTH domain that disrupts binding to m6A and found that the onset of meiosis was delayed in this strain compared to control. Both data support our findings that Pho92 promotes meiotic progression via m6A. These data are included as Figure 3E and Figure 3—figure supplement 2A and 2B respectively.

Reviewer #2:Varier et al. identified Pho92 as an m6A reader protein using differentially labeling followed by mass spec in this manuscript. They revealed that Pho92 associates with the 3'end of meiotic mRNAs in an m6A-dependent and independent manner using CLIP-seq. They show that Pho92 interacts with Paf1C in the nucleolus while it promotes m6A-dependent mRNA decay via the CCR4-NOT complex. They suggested a model that m6A enhances protein synthesis and mRNA decay to facilitate meiosis. The function of m6A in *Saccharomyces cerevisiae* is much less known than in other species. Identifying a new reader protein and providing insights into its possible biological functions are innovative and significant to the field. The authors also combined a battery of cell biology, biochemical, and sequencing approaches to provide convincing conclusions that Pho92 is an m6A reader and its possible role in RNA decay.• The authors need to revise m6A as N6-methyladenosine (m6A).

We have now revised m6A in the title as *N6*-methyladenosine (m6A).

• The authors wrote "m6A modified (GGAmCU) or unmodified (GGACU)" which might be misleading. Am is likely interpreted as 2'O methylation, but I think the authors meant methylation in the base.

We agree and corrected this. It now reads GGm6ACU.

• Identifying Pho92 as an m6A reader is a success; it is a YTH protein homolog that makes the discovery very convincing together with all the sequencing and biochemical data. However, the authors claimed that Pho92 is likely the only m6A reader, which could be right or wrong. YTHDF and YTHDC proteins have been considered the "only" m6A readers in humans for a few years, but later more "m6A readers" were identified. Pho92 did not completely cover IME2 opens the possibility of additional m6A readers.

We thank the reviewer for pointing this out. We have therefore removed this claim in the text. We have written the following header linked to figure 1 instead:

“Pho92/Mrb1 is a developmentally regulated m6A reader in yeast”

• Figure 3A, the authors could compare human METTL3/14 data with YTHDF proteins to compare with IME4 overlap with Pho92.

We agree that such an analysis would be intriguing. However, to this date there is no systematic analysis of CLIP with YTH m6A reader proteins in the presence of absence of m6A (e.g. METTL3 KO or METTL14 KO). Another confounding factor for the analysis is that there are three YTHDF proteins in mammalian cells, and it remains somewhat controversial whether they act redundantly, or each have different functions. Noteworthily, there is evidence that the YTH reader protein YTHDC2 functions in an m6A independent way, suggesting m6A independent binding to RNA is conserved (Li et al. 2022). A systematic analysis is needed to see whether YTHDF proteins can associate with RNA in m6A independent manner across different species.

• Can the authors comment on why only 30% of m6A sites are consistent with published data sites?

We thank the reviewer for the comment and were intrigued about this as well. We think there are several reasons why this could be the case. First, the published datasets encompass several timepoints throughout yeast gametogenesis. For our analysis, we only collected data from a single timepoint. Second, there are substantial technical differences between the protocols. For example, we performed a miCLIP (m6A antibody + UV crosslinking), while the published data used m6A IP followed by sequencing or an antibody independent method (MAZTER-seq) (Schwartz et al. 2013; Garcia-Campos et al. 2019). Third, we think that our miCLIP dataset is more enriched for transcripts that are well expressed and is possibly less sensitive for transcripts that are relative lowly expressed. Regarding this point we have now included a figure in the manuscript (Figure 1 —figure supplement 2C) and added the following statement in the manuscript.

“We noticed that m6A modified transcripts detected by miCLIP showed higher expression levels compared to m6A transcripts identified in the published dataset, which could explain the difference overlap between the two datasets (Figure 1 —figure supplement 2C).”

• Can the authors provide the overlap percentage of m6A sites identified using miCLIP and ime4-dependent sites?

We thank the reviewer for the suggestion. We were not completely sure which comparisons were meant by the reviewer, but we assume that it could be either of the following:

A comparison of miCLIP identified m6A sites with ime4-dependent sites. To clarify the m6A sites were identified by determining the Ime4-dependent, and thus we used a combination of WT and *ime4*∆ mutant miCLIP experiments. In yeast, Ime4 is essential for all m6A methylation, hence we used the *ime4*∆ mutant for the miCLIP as the negative control. Given that the m6A antibody crosslinks to RNA in a non-specific manner as well as, we used the *ime4*∆ mutant data to identify the Ime4-dependent peaks. Thus, per definition the m6A identified sites are ime4-dependent. We went over the manuscript and clarified this where needed. Below a further detailed explanation:

In the manuscript we state: “To identify m6A sites, we filtered for miCLIP peaks that were significantly reduced in *ime4*∆ compared to wild-type cells (log2FoldChange <= -1, adjusted p value < 0.001), and found 1286 m6A sites in 870 genes”. It is worth noting that the total number of miCLIP peaks was 9519 in the WT (both Ime4 dependent and independent).”

Approximately 14% of miCLIP peaks could be assigned as m6A sites because their signal was reduced in *ime4*∆ compared to WT. Another study made comparable observations when using the miCLIP2 technique. In this study more than 500,000 peaks in WT mESCs were identified, and this was narrowed down to 11,707 m6A sites based on Mettl3dependence (Kortel et al. 2021).

The second option is that reviewer referred to the overlap between m6A sites identified and Pho92 Ime4 dependent sites. This information is provided in Figure 2C. Here in the Venn diagram we see that 281/642 Ime4-dependent Pho92 binding sites overlap with an m6A site as defined by our miCLIP.

• The author claims that Paf1C contributes to the localization of Pho92 to the nucleus, and m6A modified transcripts facilitate the transition of their reader Pho92 into the cytoplasm. This conclusion is not supported, there are many other possibilities.

We thank the reviewer for the comment. Our data are consistent with a model that Paf1C directs Pho92 to mRNAs co-transcriptionally. First, Pho92 localization to nucleus is reduced in *leo1*∆ mutant, and Paf1 depletion mutant. Second, Pho92 ChIP signal is enriched at several Pho92 targets in Leo1 dependent manner. We agree that a functional link is lacking in our data. For example, we tried to determine whether Pho92 binding to m6A transcripts was reduced in Paf1C mutants, but this was technically challenging.

In addition to our model, we have now also discussed other possibilities. For example, it is possible that Pho92 has a second role in nucleus to degrade RNA there, and thereby could regulate chromatin and transcription. A nuclear function has been reported for other YTH proteins. However, our data suggest that m6A modified transcripts are degraded in mostly a translation dependent manner. Another possibility is that Pho92 has an m6A independent role in the nucleus. We agree that more work is needed to dissect this further. Nevertheless, we think it is intriguing that Pho92 is dynamically localized, and that the data are consistent with the model that Pho92 is recruited co-transcriptionally. We added the following section to result section.

“Our data are consistent with a model where Pho92 is loaded co-transcriptionally to nascently produced mRNAs. However, it is also possible that Pho92 has a regulatory function in the nucleus involving chromatin and transcription as has been reported for other YTH domain containing proteins. “

• Pho92 associates with nascent nuclear RNA in a Paf1C-dependent manner, however, what is the function of Pho92 interacting with nascent RNA? Do the authors hypothesize Pho92 works like YTHDC1, which facilitate m6A translocation and m6A-dependent splicing? These data on Paf1C and Pho92 are floating without conclusive function as it is currently presented.

We are thankful for the comment. We acknowledge that we have not identified a function for the Paf1C mediated localization of Pho92 to target genes. We have spent quite some time trying to link the Paf1C interaction of Pho92 to function, however this turned out to be technically challenging. We think that the link between Paf1C and Pho92 nuclear localization and Pho92 binding ORFs of target genes suggests that Paf1C can direct Pho92 to target transcript co-transcriptionally. Whether possibly splicing is regulated by Pho92 or other nuclear processes remains to be determined. We have now included a discussion for the other possibilities, and added the following section:

“Another possibility is that Pho92 has a separate regulatory function in the nucleus involving chromatin and transcription as has been reported for other YTH domain containing proteins.”

• The impact of Pho92 on RNA decay is very convincing. However, the impact on translation is less compelling. The differences are tiny in protein level (Figure 6H), which was similarly seen in human YTHDF1 (promotes translation under stimuli)

We agree that the effect of Pho92 on mRNA decay is substantial whilst the impact on protein synthesis is less profound. Nevertheless, what was striking is that protein levels of Pho92/m6A targets do not follow the mRNAs as determined by proteomics and by time course experiments of Pho92 targets. This suggests that protein synthesis is at least in part affected in the *pho92*∆ mutant. Given that Pho92 is also at active ribosomes, and Pho92 targets are linked with increased translation efficiency, we think Pho92 roles in decay may be linked to controlling translation as well.

• Overall, it is a great study and provided many lines of useful information. However, this paper is trying to say that Pho92 covers all known human m6A readers' functions. The authors might consider organizing and emphasizing the best data.

In agreement with the suggestions of the reviewer, we have now weakened the interpretation of the translation data and emphasize more on the fact that our observation is decay in a translation-dependent manner. However, the transcripts that are expressed more are clearly not expressed more at the protein level, which could be due to impaired protein synthesis.

Reviewer #3:1. By eye it is difficult to appreciate the re-localization of Pho92 in the cytoplasm after IME1 induction (Figure 4D). Furthermore, the data is not shown for the different mutant conditions (only the quantification is shown). This system is rather artificial as Pho92 is normally not expressed at 0h and 2h after induction. Could the authors instead check the localization of endogenous Pho92? By staining or fractionation experiment?

As suggested by the reviewer, we have now included representative images from a *leo1*∆ alongside WT in Figure 4D. We have also determined the localization of endogenous Pho92 and have added the images and quantification in Figure 4—figure supplement 2A and 2B.

2. The decreased binding of Pho92 to chromatin upon leo1 depletion may not be related to their direct interaction. Another explanation could be that leo1 affects transcription, and therefore less m6A modified transcripts would be produced, which would decrease the binding of Pho92 to chromatin. To discard this possibility the authors should check the RNA level upon leo1 depletion, and also check whether the binding of Pho92 is independent of the nascent transcripts. I also note that the decreased binding is not supported by statistics

In accordance with the reviewers’ comments, to discard the possibility that *leo1*∆ affects transcription resulting in less production of m6A modified transcripts thereby decreasing the binding of Pho92, we present RNA levels of *BDF2*, *GUT2* and protein levels of Pho92 in Figure 4- figure-supplement 3B and 3C. Lastly, Pho92 binding observed in ChIP experiments is independent of RNA as the ChIP samples were treated with RNase. We have now included a statement in the methods specifying this. Decrease in Pho92 binding observed in ChIP experiments is now supported by data from 3 repeats and relevant statistics.

3. It is unusual to quantify the level of m6A instead of the transcript itself to demonstrate a role in mRNA decay. If the effect is visible at the m6A level it should also be visible at the transcript level. I am a bit confused and not convinced with this indirect approach.

The standard method for measuring mRNA decay and mRNA half-lives is by shutting down transcription and follow the mRNA transcripts levels. In this assay, we did the same and we followed the fate of the m6A mRNAs relative to the fate of all mRNAs. Thus, our method does not deviate much from a standard mRNA decay assay. The reasons for the approach are outlined in the manuscript. One reason is that it is not clear what fraction of target transcripts are m6A modified, which can potentially mask effects on decay. In addition, the thiolutin concentration used will likely not shut down transcription completely, which would prove problematic for measuring absolute mRNA decay rates but not for our relative measurements.

To address the reviewer’s suggestion, we have performed a complementary approach and determined mRNA half-live measurements of Pho92 target transcripts in wild type and *pho92*∆ cells. To achieve a rapid transcriptional shut down of meiotic transcripts, we switched cells to a nutrient rich condition (return to growth). We observed that the mRNA half-life of meiotic transcripts was short (in the minute range) and that in the *pho92*∆ mutant the mRNA half-lives were increased by out 1.5-fold. These data are included in Figure 5D and Figure 5—figure supplement 1E. Together, this makes a case that Pho92 controls the mRNA decay of m6A modified transcripts.

References:

Garcia-Campos MA, Edelheit S, Toth U, Safra M, Shachar R, Viukov S, Winkler R, Nir R, Lasman L, Brandis A et al. 2019. Deciphering the "m(6)A Code" via AntibodyIndependent Quantitative Profiling. *Cell* 178: 731-747 e716.

Kortel N, Ruckle C, Zhou Y, Busch A, Hoch-Kraft P, Sutandy FXR, Haase J, Pradhan M, Musheev M, Ostareck D et al. 2021. Deep and accurate detection of m6A RNA modifications using miCLIP2 and m6Aboost machine learning. *Nucleic Acids Res* 49: e92.

Li L, Krasnykov K, Homolka D, Gos P, Mendel M, Fish RJ, Pandey RR, Pillai RS. 2022. The XRN1-regulated RNA helicase activity of YTHDC2 ensures mouse fertility independently of m(6)A recognition. *Mol Cell* 82: 1678-1690 e1612.

Schwartz S, Agarwala SD, Mumbach MR, Jovanovic M, Mertins P, Shishkin A, Tabach Y, Mikkelsen TS, Satija R, Ruvkun G et al. 2013. High-resolution mapping reveals a conserved, widespread, dynamic mRNA methylation program in yeast meiosis. *Cell* 155: 1409-1421.