# Supplementary figures and images for: N6-methyladenosine (m6A) reader Pho92 is recruited co-transcriptionally and couples translation to mRNA decay to promote meiotic fitness in yeast
Source: eLife. 2022 Nov 24;11:e84034. doi: 10.7554/eLife.84034 (PMC9731578; doi:10.7554/eLife.84034)

Hxk1  
(red)

Pho92  
(green)

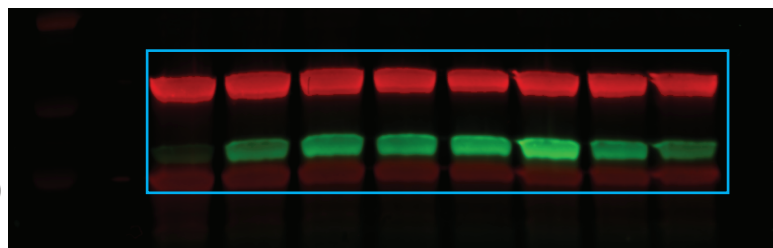

Supplement: Figure 1—source data 1. [file elife-84034-fig1-data1.zip › Figure 1 - source data 1.pdf]

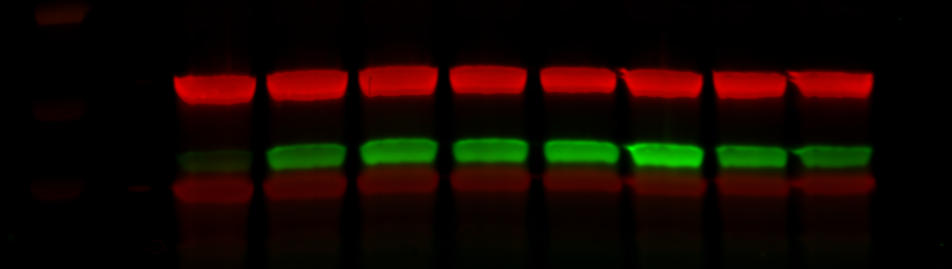

Supplement: Figure 1—source data 1. [file elife-84034-fig1-data1.zip › Figure 1 - source data 1.tif]

Hxk1  
(red)  
Pho92  
(green)

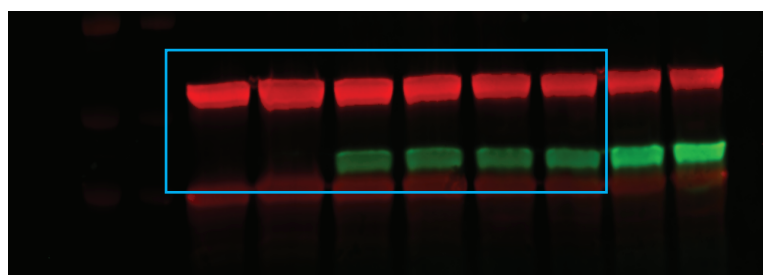

Supplement: Figure 1—source data 2. [file elife-84034-fig1-data2.zip › Figure 1 - source data 2.pdf]

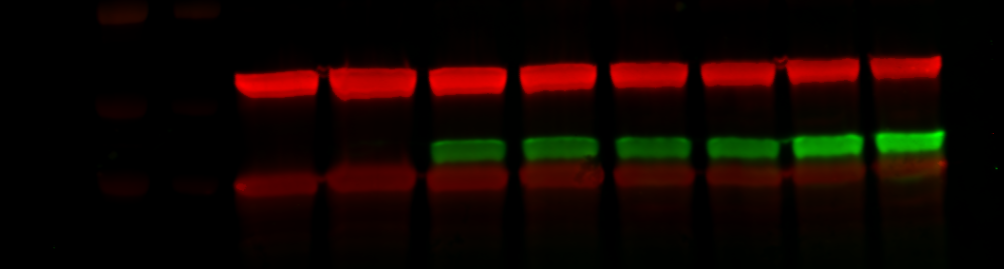

Supplement: Figure 1—source data 2. [file elife-84034-fig1-data2.zip › Figure 1 - source data 2.tif]

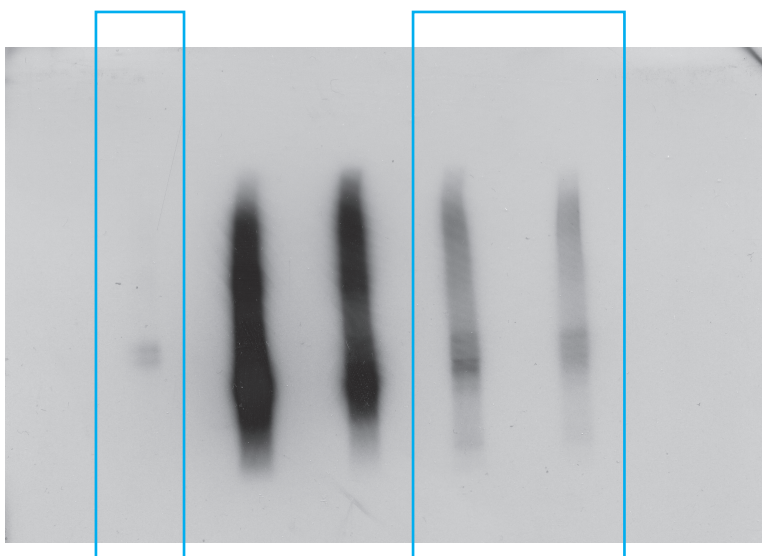

Supplement: Figure 1—source data 3. [file elife-84034-fig1-data3.zip › Figure 1 - source data 3.pdf]

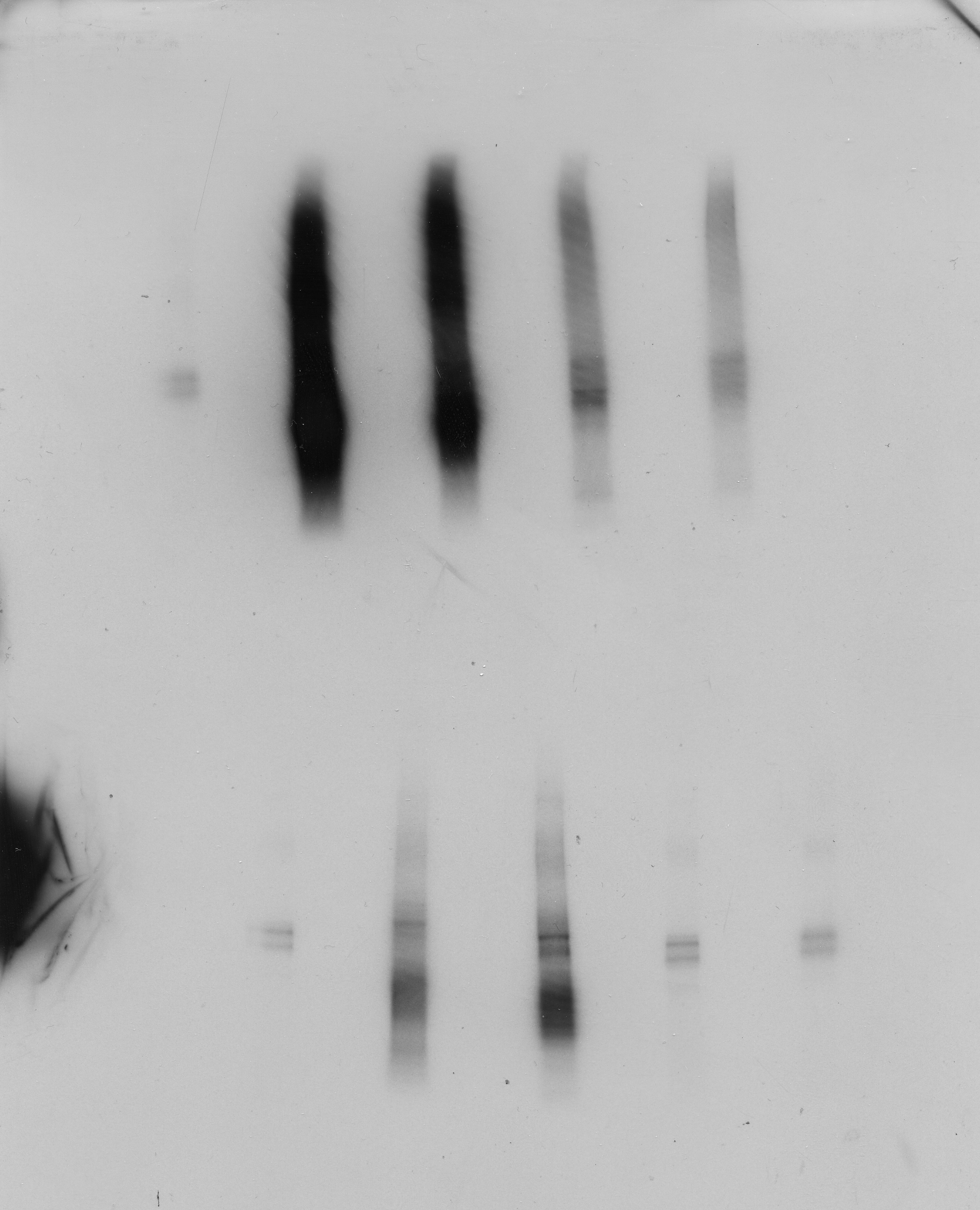

Supplement: Figure 1—source data 3. [file elife-84034-fig1-data3.zip › Figure 1 - source data 3.tif]

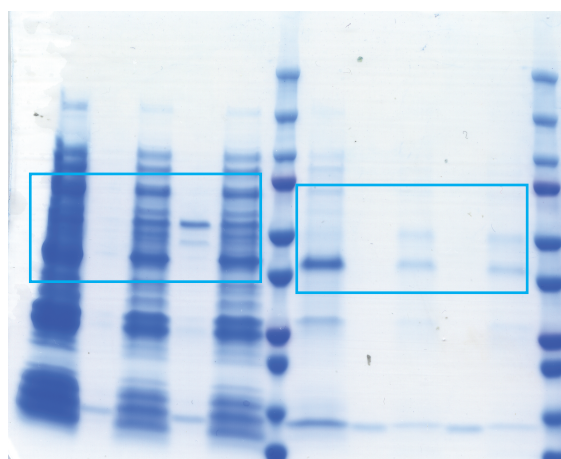

Supplement: Figure 1—figure supplement 1—source data 1. [file elife-84034-fig1-figsupp1-data1.zip › Figure 1 - figure supplement 1 - source data 1.pdf]

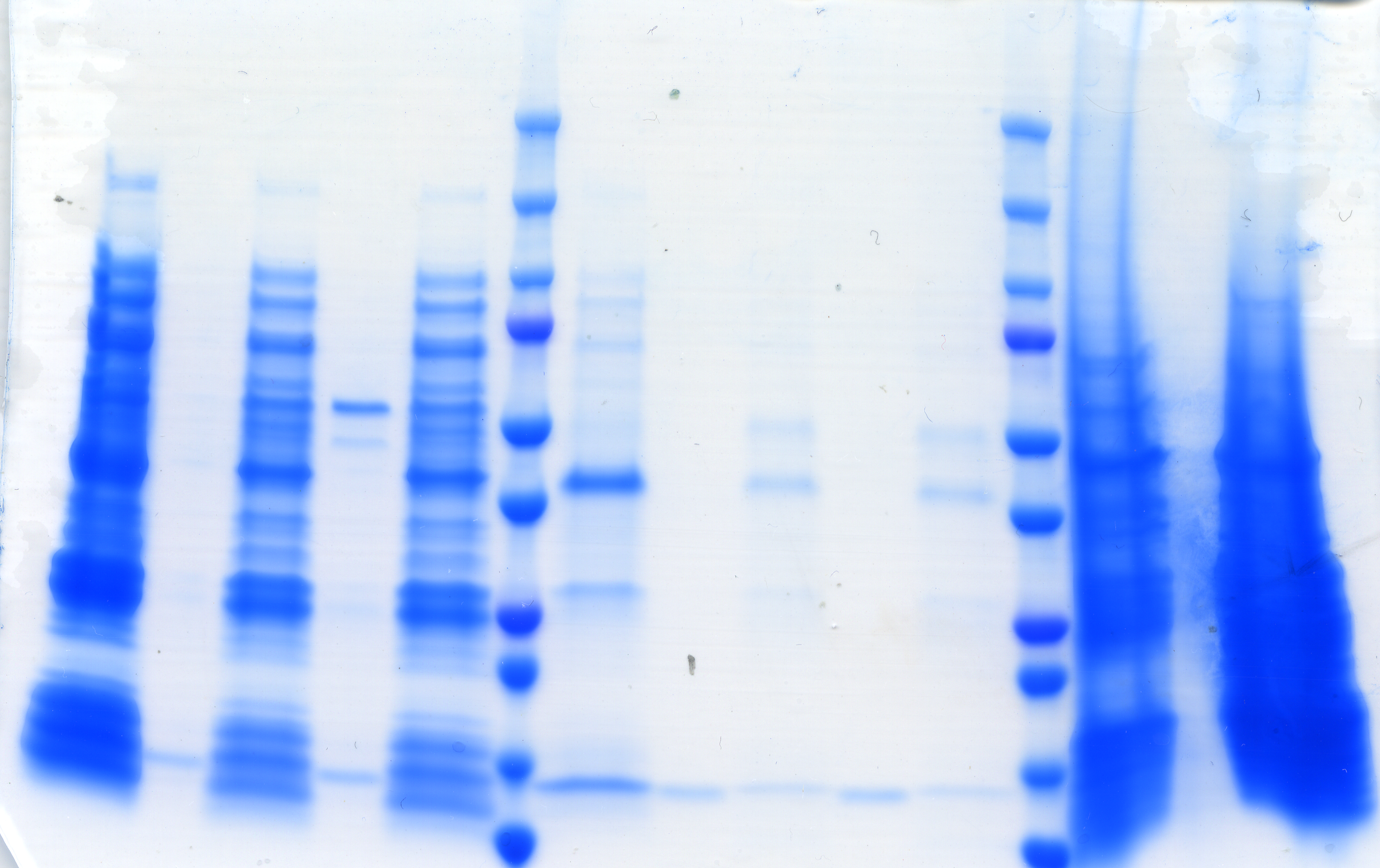

Supplement: Figure 1—figure supplement 1—source data 1. [file elife-84034-fig1-figsupp1-data1.zip › Figure 1 - figure supplement 1 - source data 1.tif]

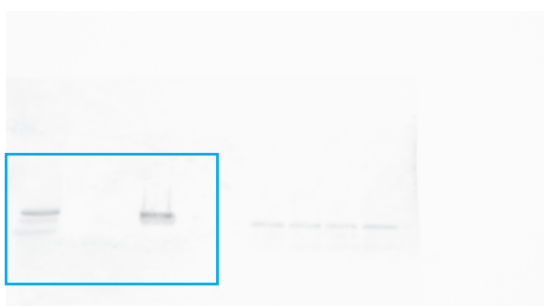

Supplement: Figure 1—figure supplement 1—source data 2. [file elife-84034-fig1-figsupp1-data2.zip › Figure 1 - figure supplement 1 - source data 2.pdf]

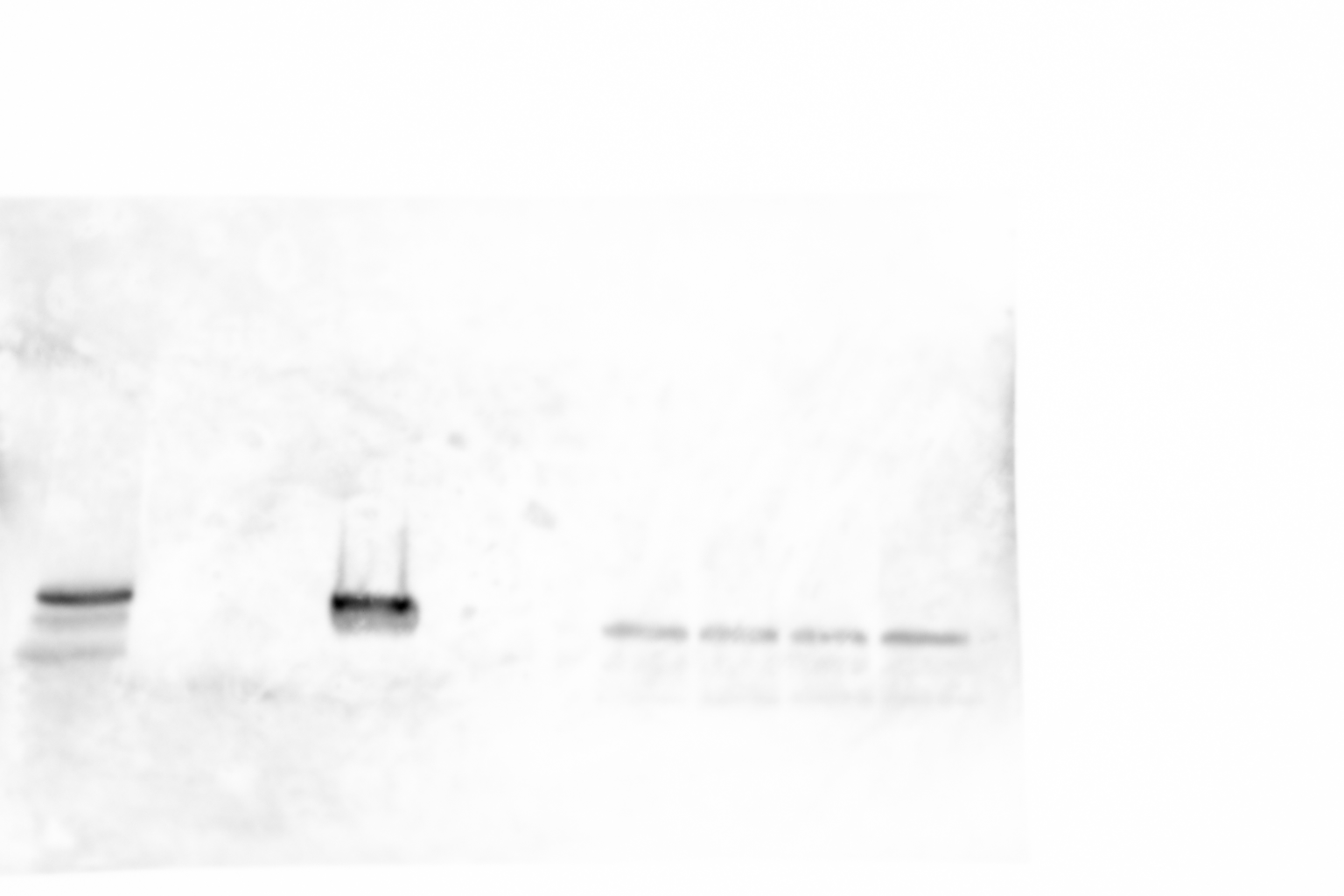

Supplement: Figure 1—figure supplement 1—source data 2. [file elife-84034-fig1-figsupp1-data2.zip › Figure 1 - figure supplement 1 - source data 2.tif]

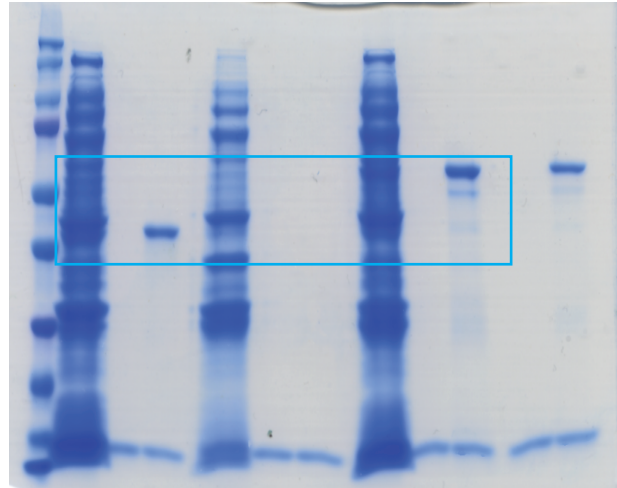

Supplement: Figure 1—figure supplement 1—source data 3. [file elife-84034-fig1-figsupp1-data3.zip › Figure 1 - figure supplement 1 - source data 3.pdf]

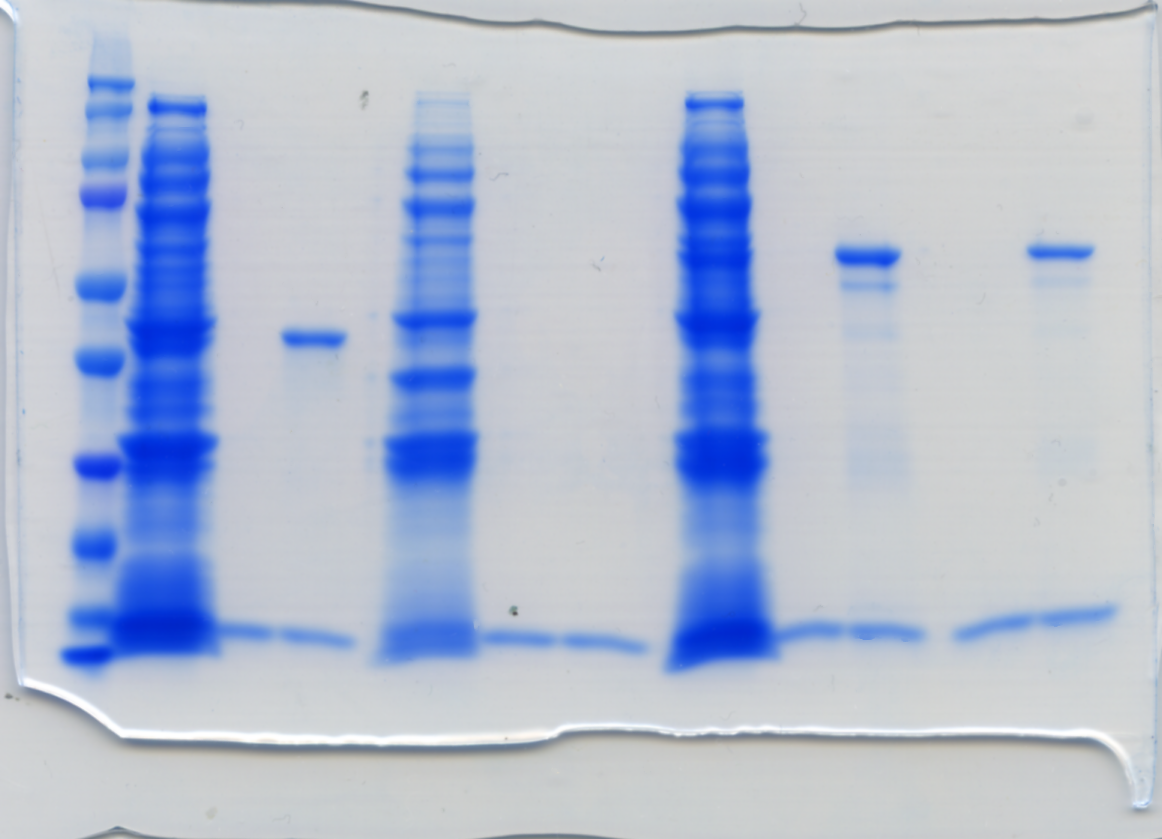

Supplement: Figure 1—figure supplement 1—source data 3. [file elife-84034-fig1-figsupp1-data3.zip › Figure 1 - figure supplement 1 - source data 3.tif]

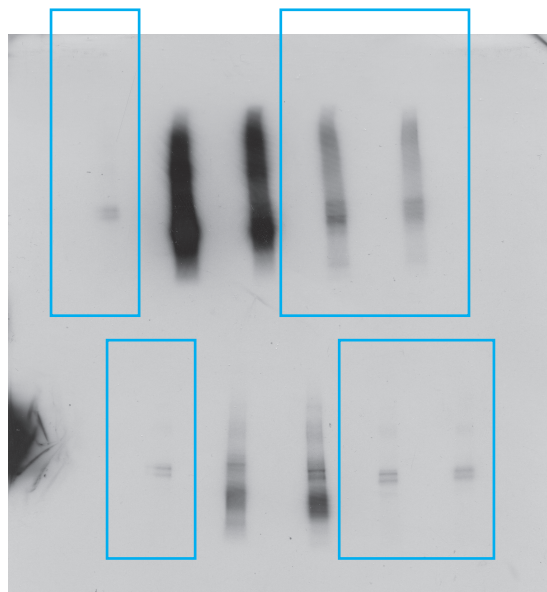

Supplement: Figure 1—figure supplement 1—source data 4. [file elife-84034-fig1-figsupp1-data4.zip › Figure 1 - figure supplement 1 - source data 4.pdf]

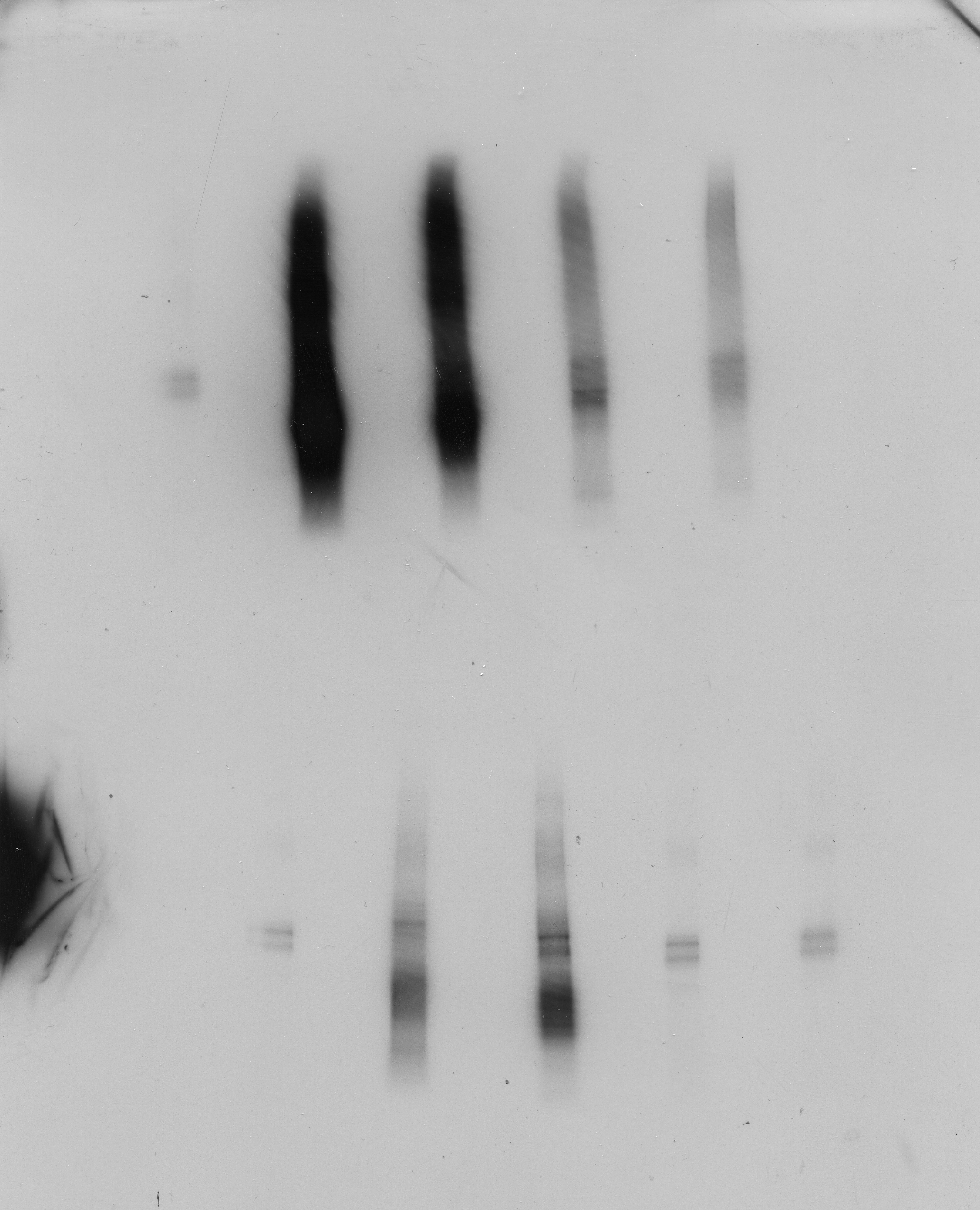

Supplement: Figure 1—figure supplement 1—source data 4. [file elife-84034-fig1-figsupp1-data4.zip › Figure 1 - figure supplement 1 - source data 4.tif]

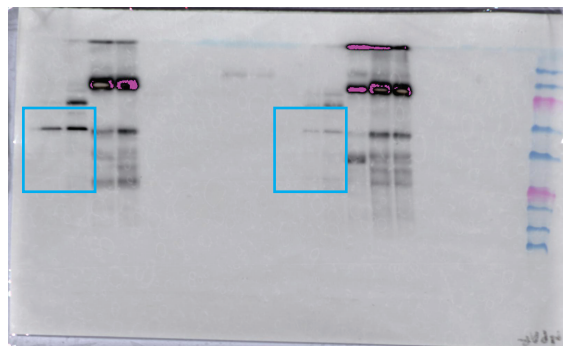

Supplement: Figure 1—figure supplement 1—source data 5. [file elife-84034-fig1-figsupp1-data5.zip › Figure 1 - figure supplement 1 - source data 5.pdf]

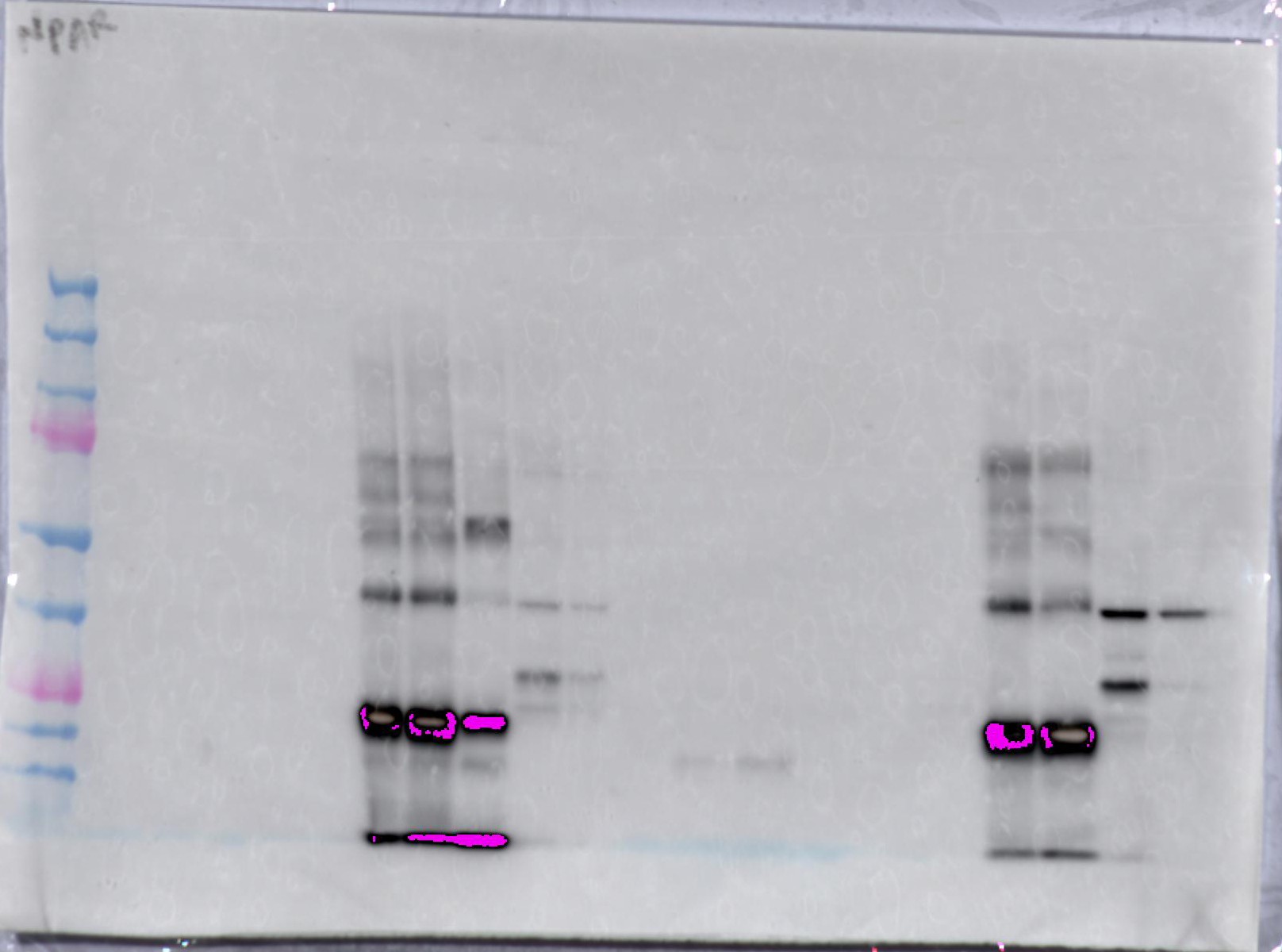

Supplement: Figure 1—figure supplement 1—source data 5. [file elife-84034-fig1-figsupp1-data5.zip › Figure 1 - figure supplement 1 - source data 5.tif]

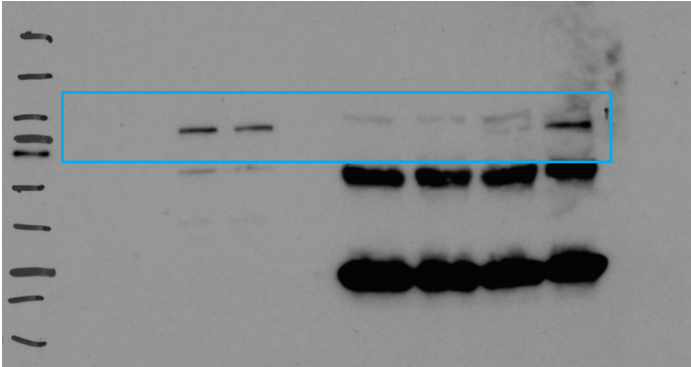

Supplement: Figure 4—source data 1. [file elife-84034-fig4-data1.zip › Figure 4 - source data 1.pdf]

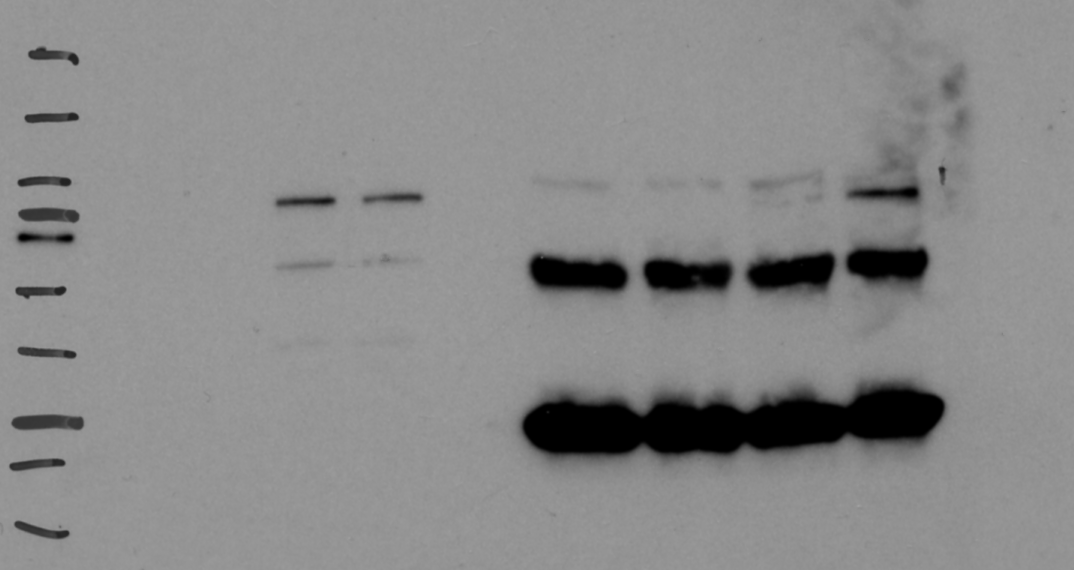

Supplement: Figure 4—source data 1. [file elife-84034-fig4-data1.zip › Figure 4 - source data 1.tif]

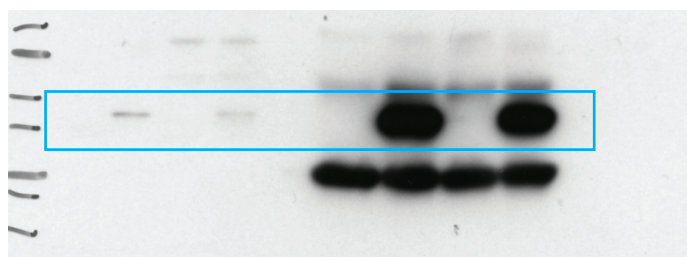

Supplement: Figure 4—source data 2. [file elife-84034-fig4-data2.zip › Figure 4 - source data 2.pdf]

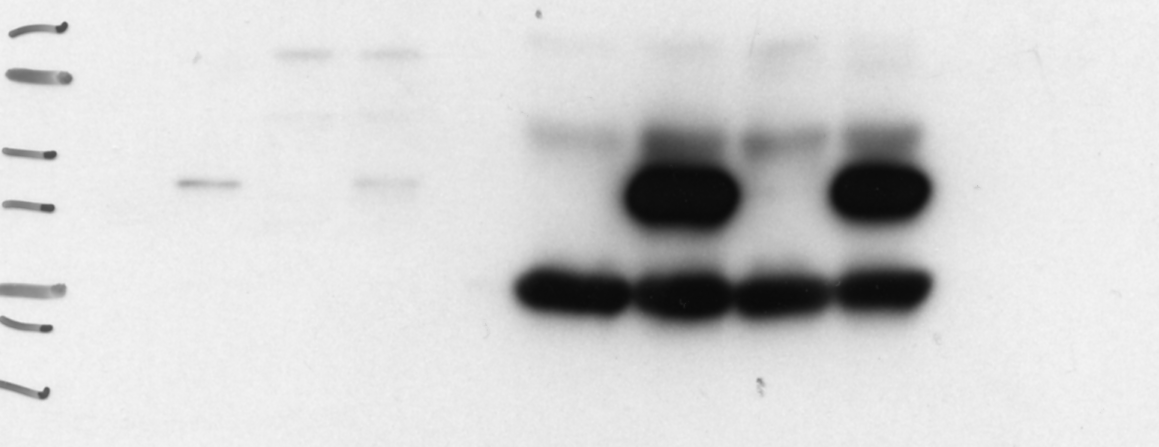

Supplement: Figure 4—source data 2. [file elife-84034-fig4-data2.zip › Figure 4 - source data 2.tif]

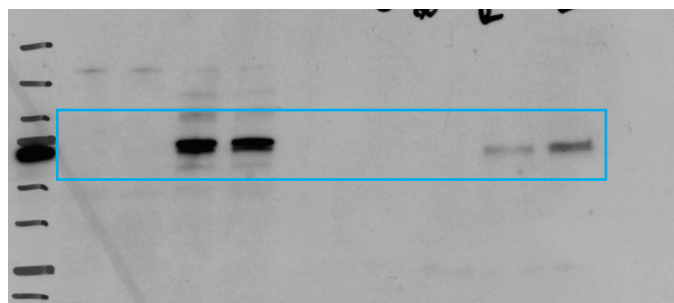

Supplement: Figure 4—source data 3. [file elife-84034-fig4-data3.zip › Figure 4 - source data 3.pdf]

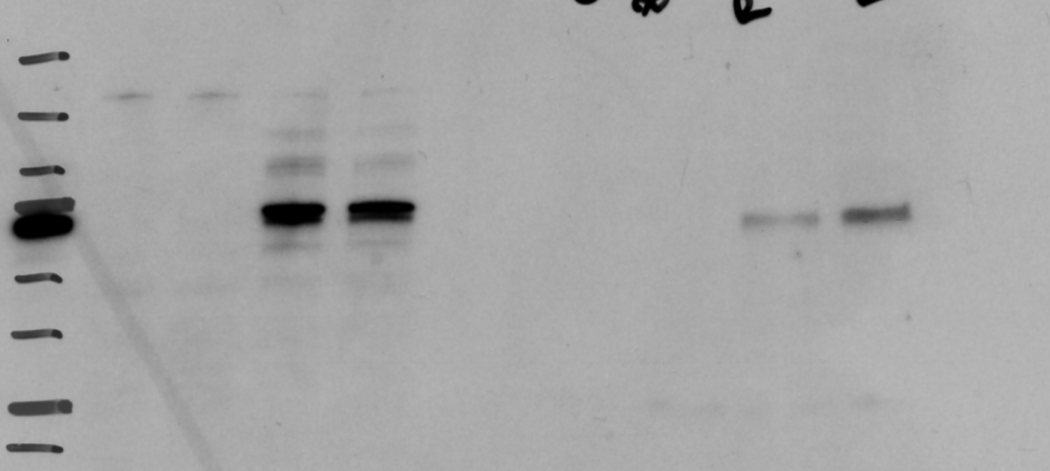

Supplement: Figure 4—source data 3. [file elife-84034-fig4-data3.zip › Figure 4 - source data 3.tif]

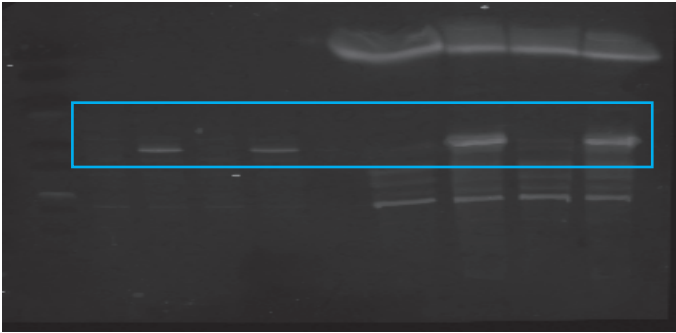

Supplement: Figure 4—source data 4. [file elife-84034-fig4-data4.zip › Figure 4 - source data 4.pdf]

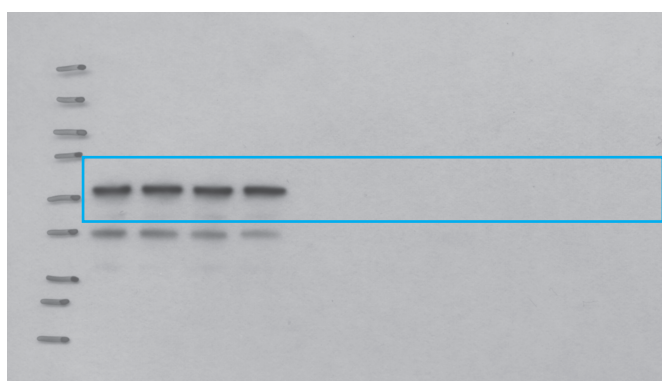

Supplement: Figure 4—source data 5. [file elife-84034-fig4-data5.zip › Figure 4 - source data 5.pdf]

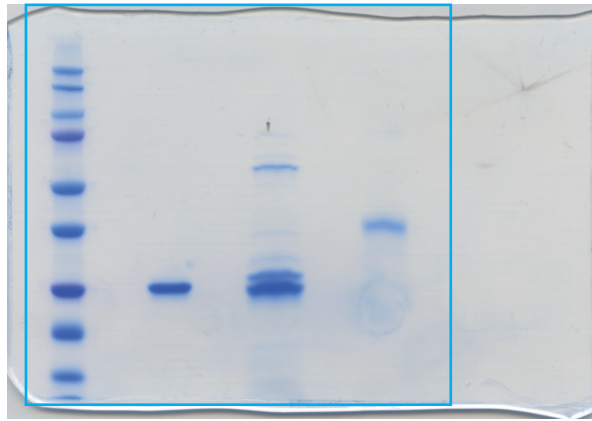

Supplement: Figure 4—source data 6. [file elife-84034-fig4-data6.zip › Figure 4 - source data 6.pdf]

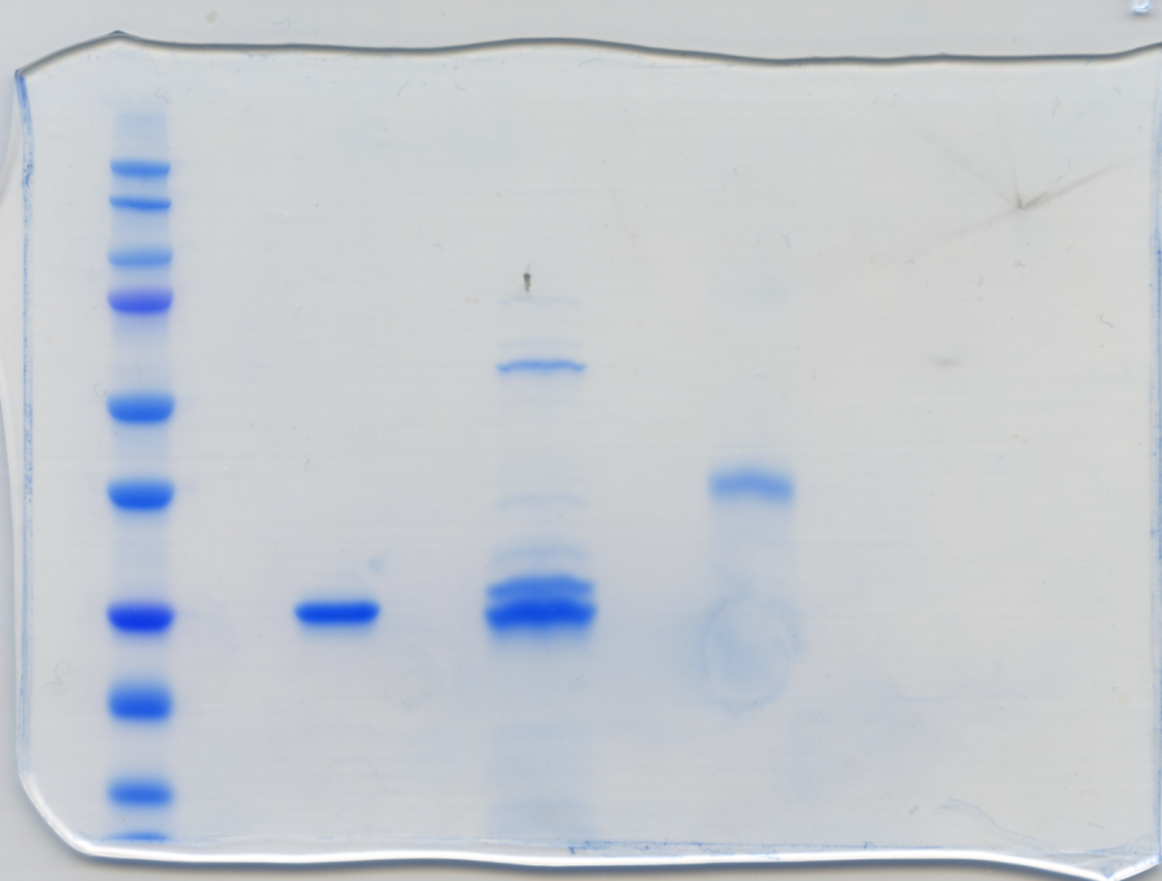

Supplement: Figure 4—source data 6. [file elife-84034-fig4-data6.zip › Figure 4 - source data 6.tif]

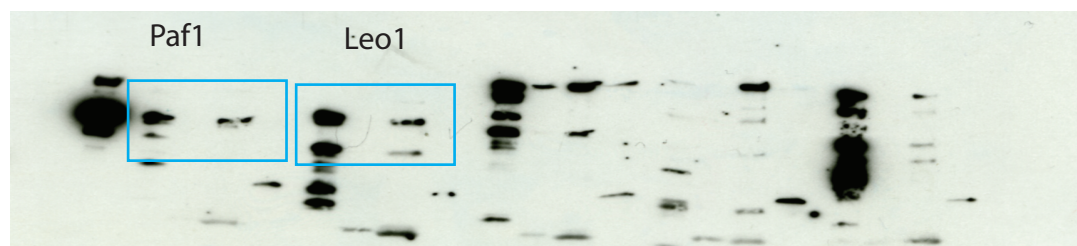

Supplement: Figure 4—source data 7. [file elife-84034-fig4-data7.zip › Figure 4 - source data 7.pdf]

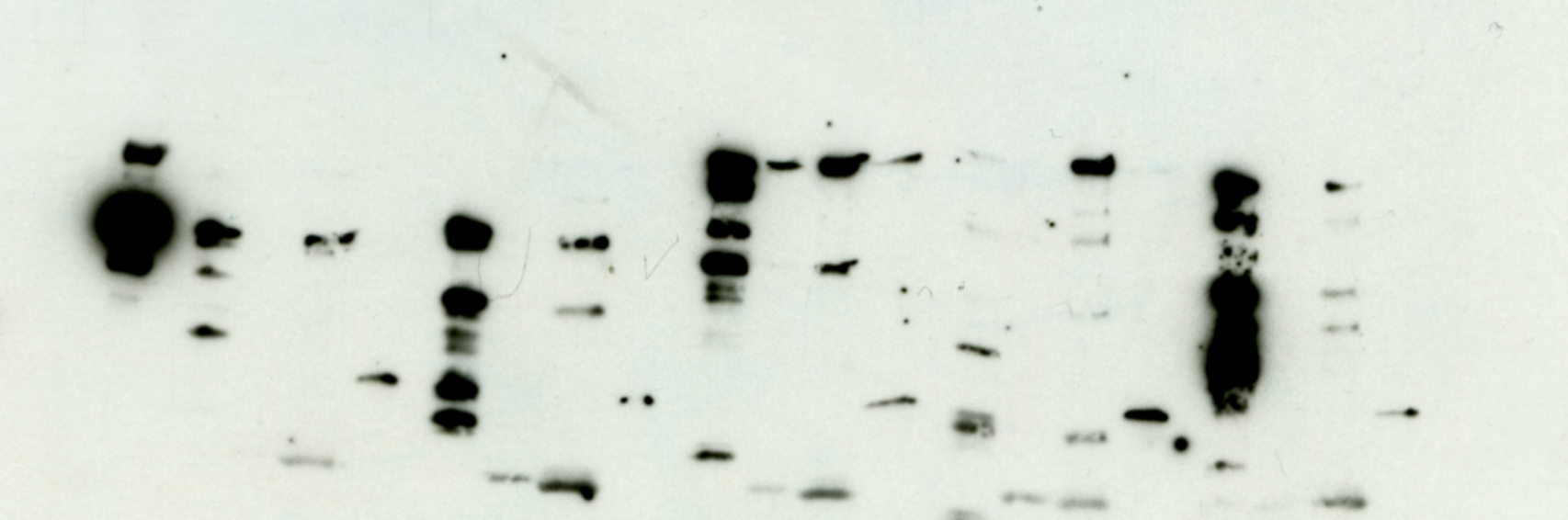

Supplement: Figure 4—source data 7. [file elife-84034-fig4-data7.zip › Figure 4 - source data 7.tif]

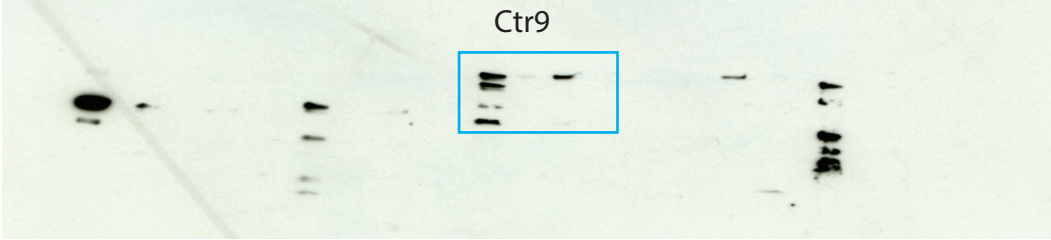

Supplement: Figure 4—source data 8. [file elife-84034-fig4-data8.zip › Figure 4 - source data 8.pdf]

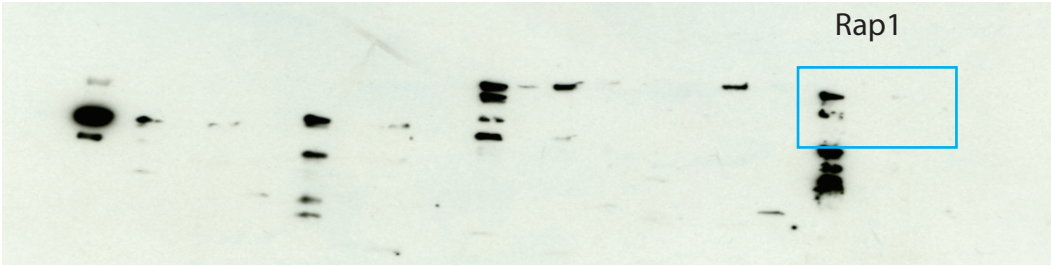

Supplement: Figure 4—source data 9. [file elife-84034-fig4-data9.zip › Figure 4 - source data 9.pdf]

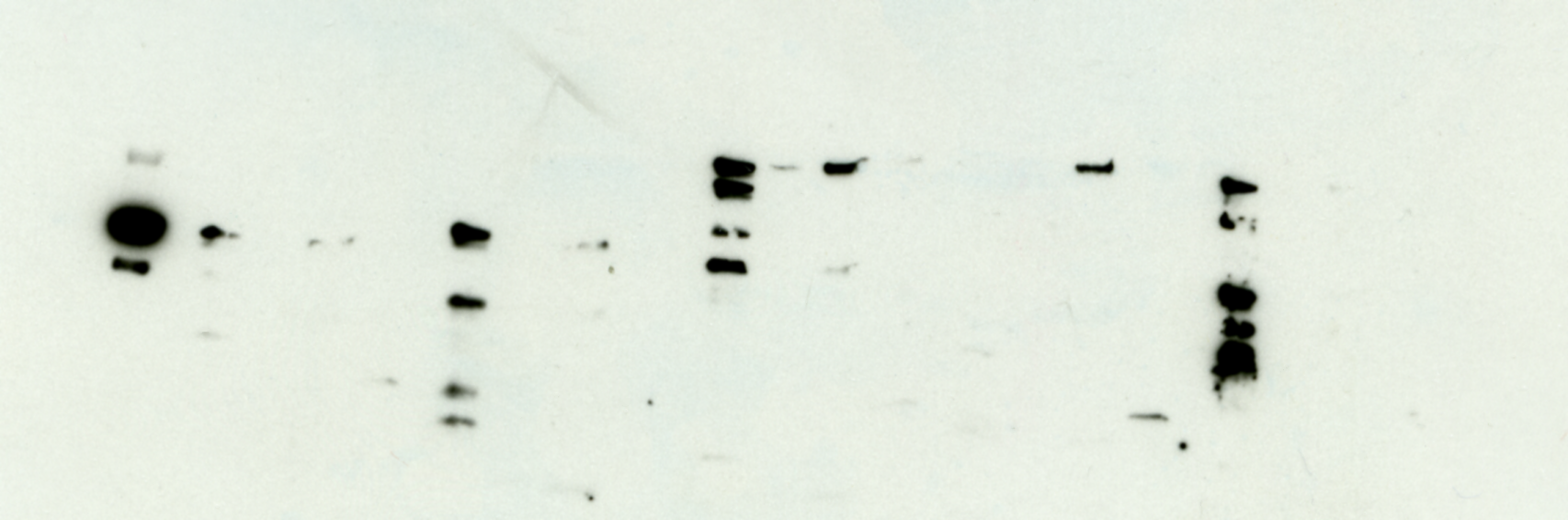

Supplement: Figure 4—source data 9. [file elife-84034-fig4-data9.zip › Figure 4 - source data 9.tif]

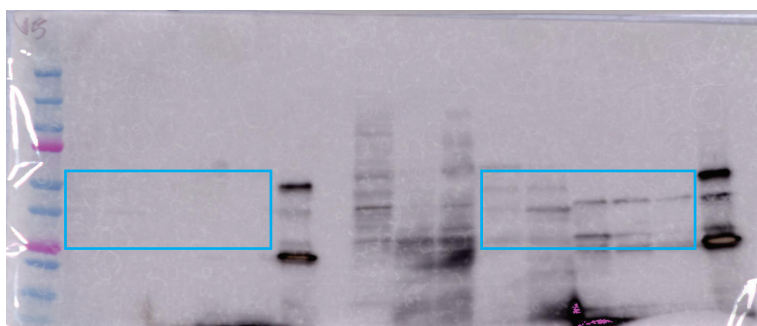

Supplement: Figure 4—figure supplement 1—source data 1. [file elife-84034-fig4-figsupp1-data1.zip › Figure 4 – figure supplement 1 - source data 1.pdf]

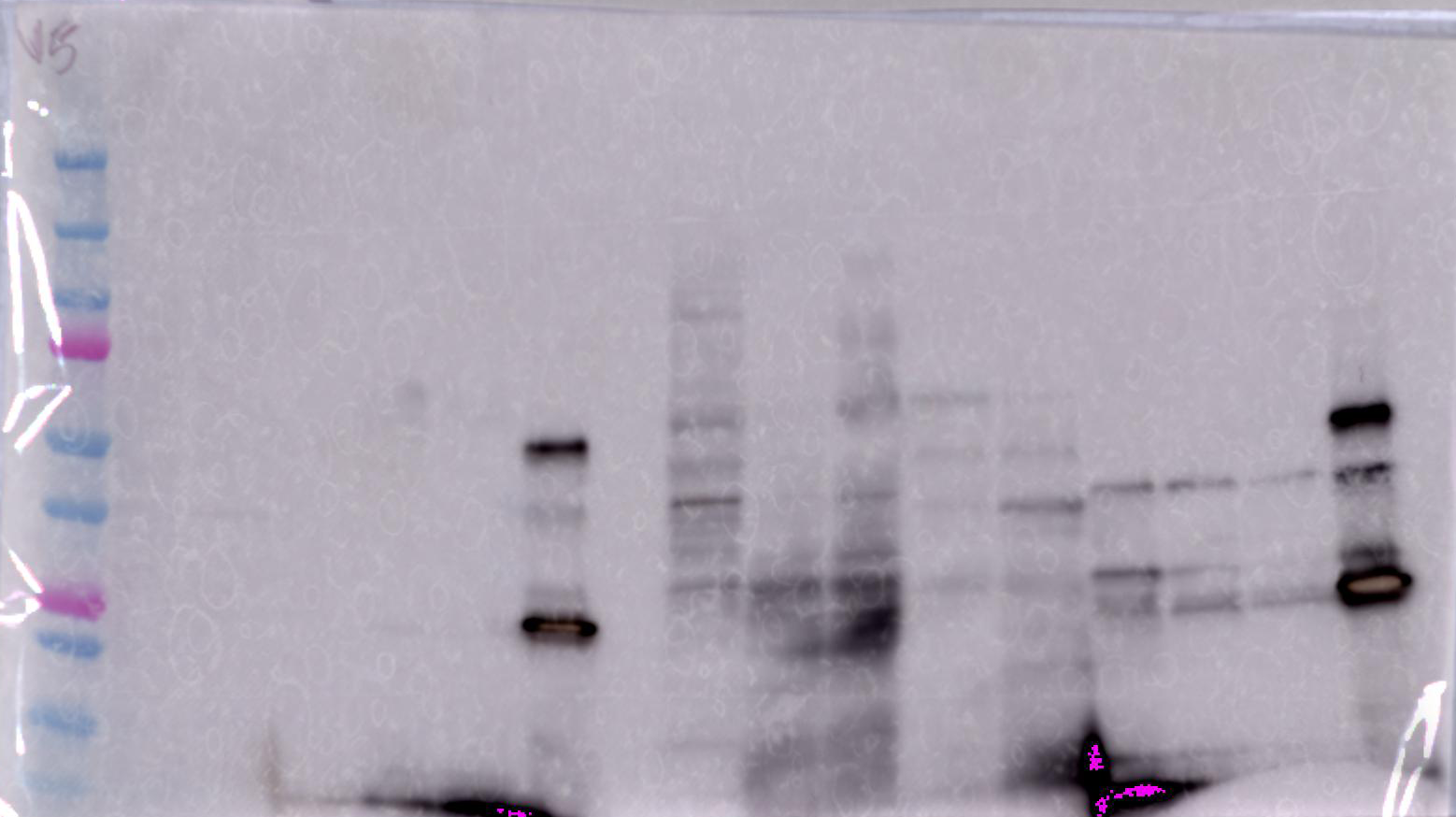

Supplement: Figure 4—figure supplement 1—source data 1. [file elife-84034-fig4-figsupp1-data1.zip › Figure 4 – figure supplement 1 - source data 1.tif]

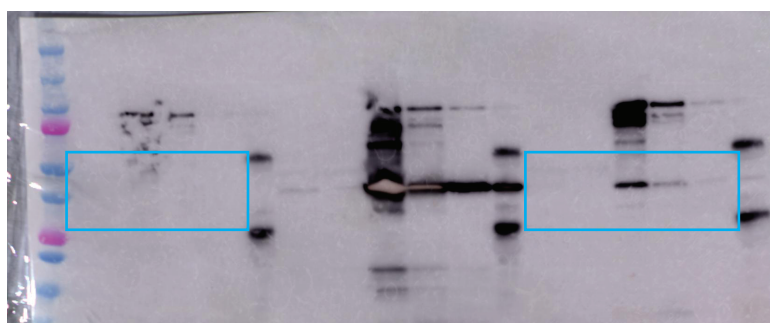

Supplement: Figure 4—figure supplement 1—source data 2. [file elife-84034-fig4-figsupp1-data2.zip › Figure 4 - figure supplement 1 - source data 2.pdf]

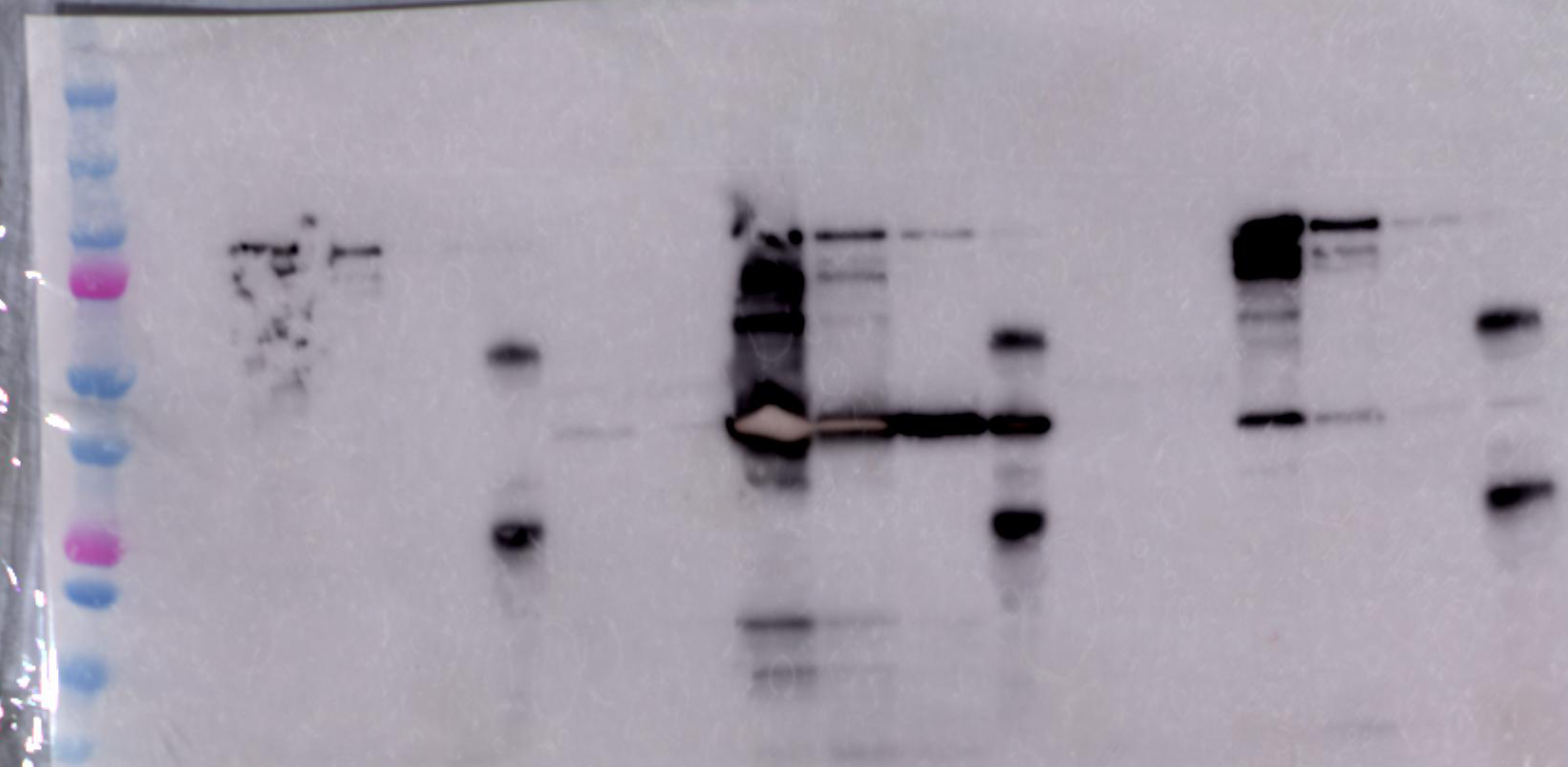

Supplement: Figure 4—figure supplement 1—source data 2. [file elife-84034-fig4-figsupp1-data2.zip › Figure 4 - figure supplement 1 - source data 2.tif]

Paf1

Hxk1

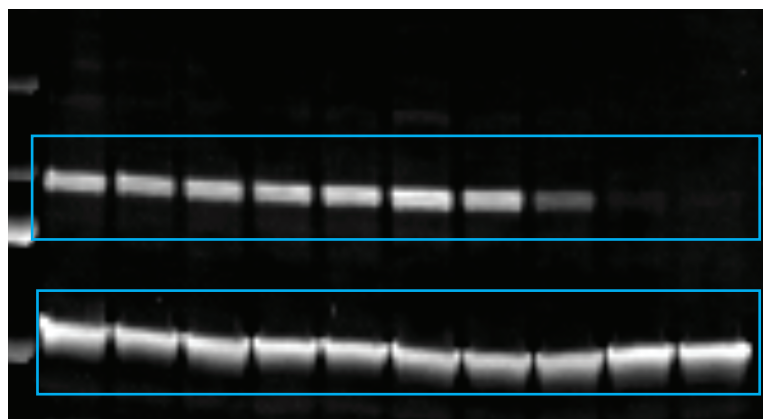

Supplement: Figure 4—figure supplement 2—source data 1. [file elife-84034-fig4-figsupp2-data1.zip › Figure 4 - figure supplement 2 - source data 1.pdf]

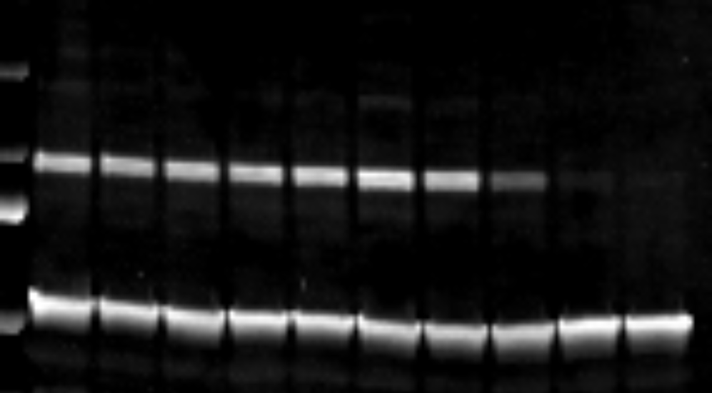

Supplement: Figure 4—figure supplement 2—source data 1. [file elife-84034-fig4-figsupp2-data1.zip › Figure 4 - figure supplement 2 - source data 1.tif]

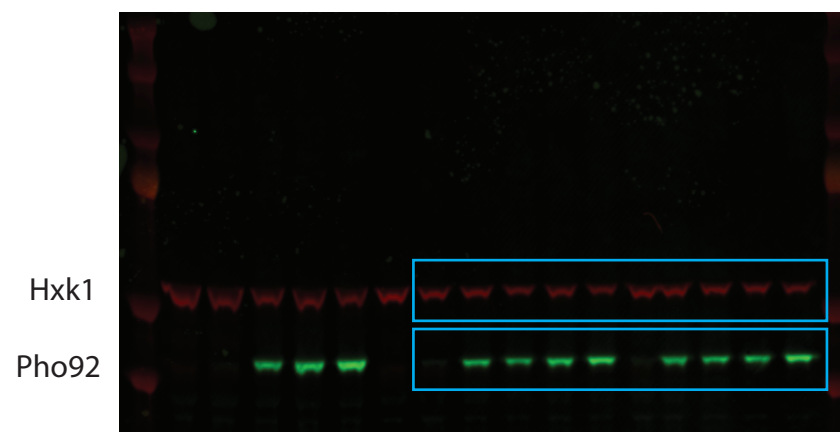

Supplement: Figure 4—figure supplement 3—source data 1. [file elife-84034-fig4-figsupp3-data1.zip › Figure 4 - figure supplement 3 - source data 1.pdf]

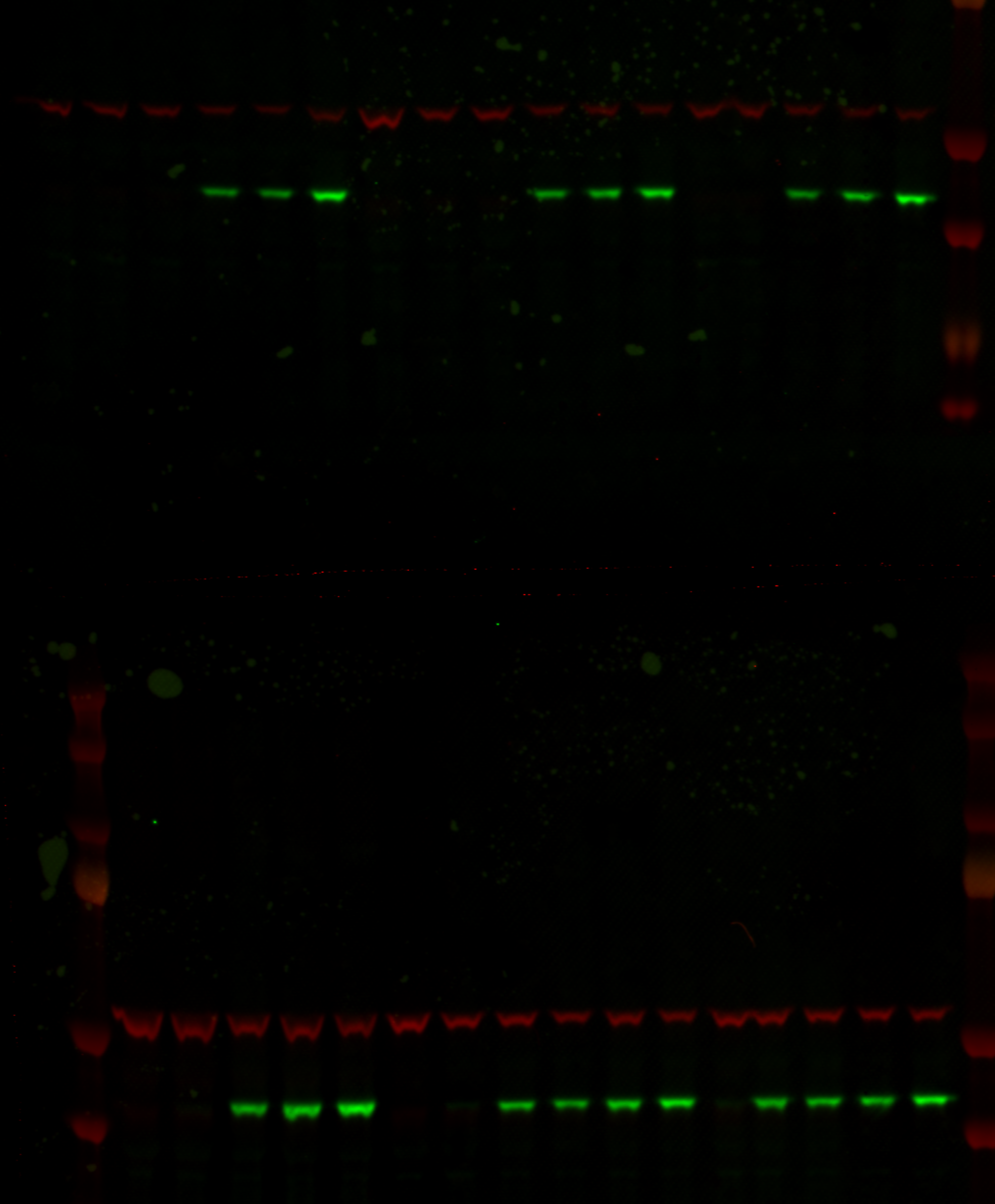

Supplement: Figure 4—figure supplement 3—source data 1. [file elife-84034-fig4-figsupp3-data1.zip › Figure 4 - figure supplement 3 - source data 1.tif]

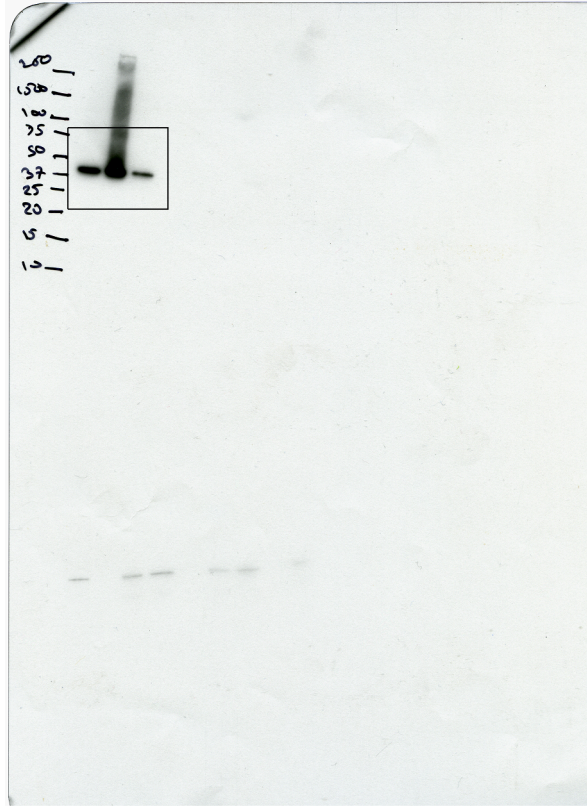

Supplement: Figure 6—source data 1. [file elife-84034-fig6-data1.zip › Figure 6 - source data 1.pdf]

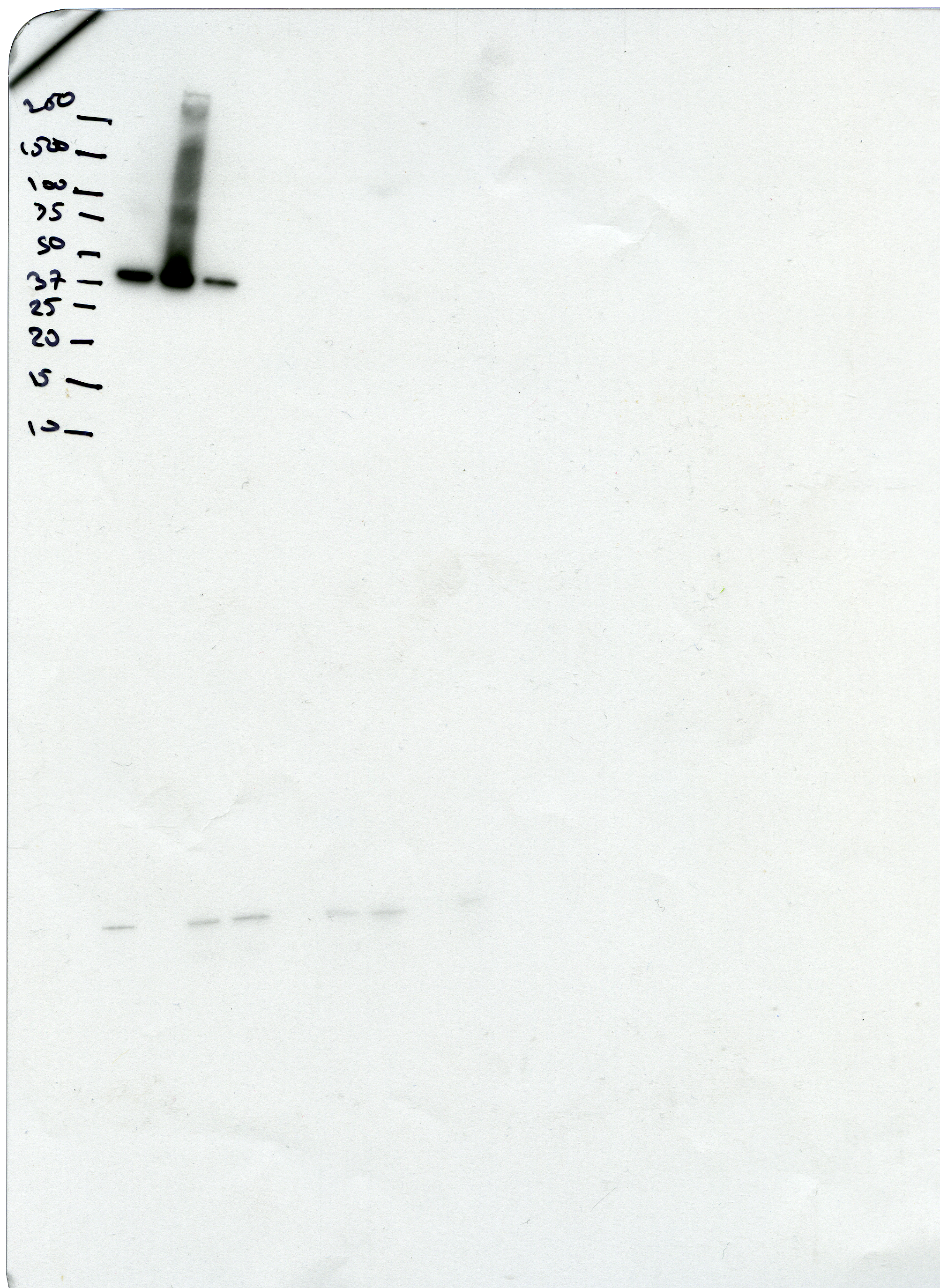

Supplement: Figure 6—source data 1. [file elife-84034-fig6-data1.zip › Figure 6 - source data 1.tif]

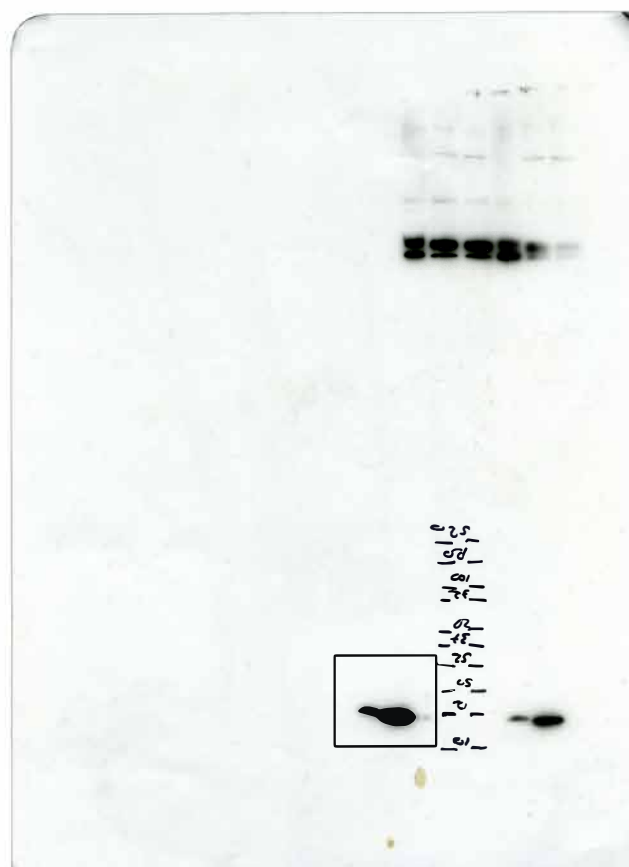

Supplement: Figure 6—source data 2. [file elife-84034-fig6-data2.zip › Figure 6 - source data 2.pdf]

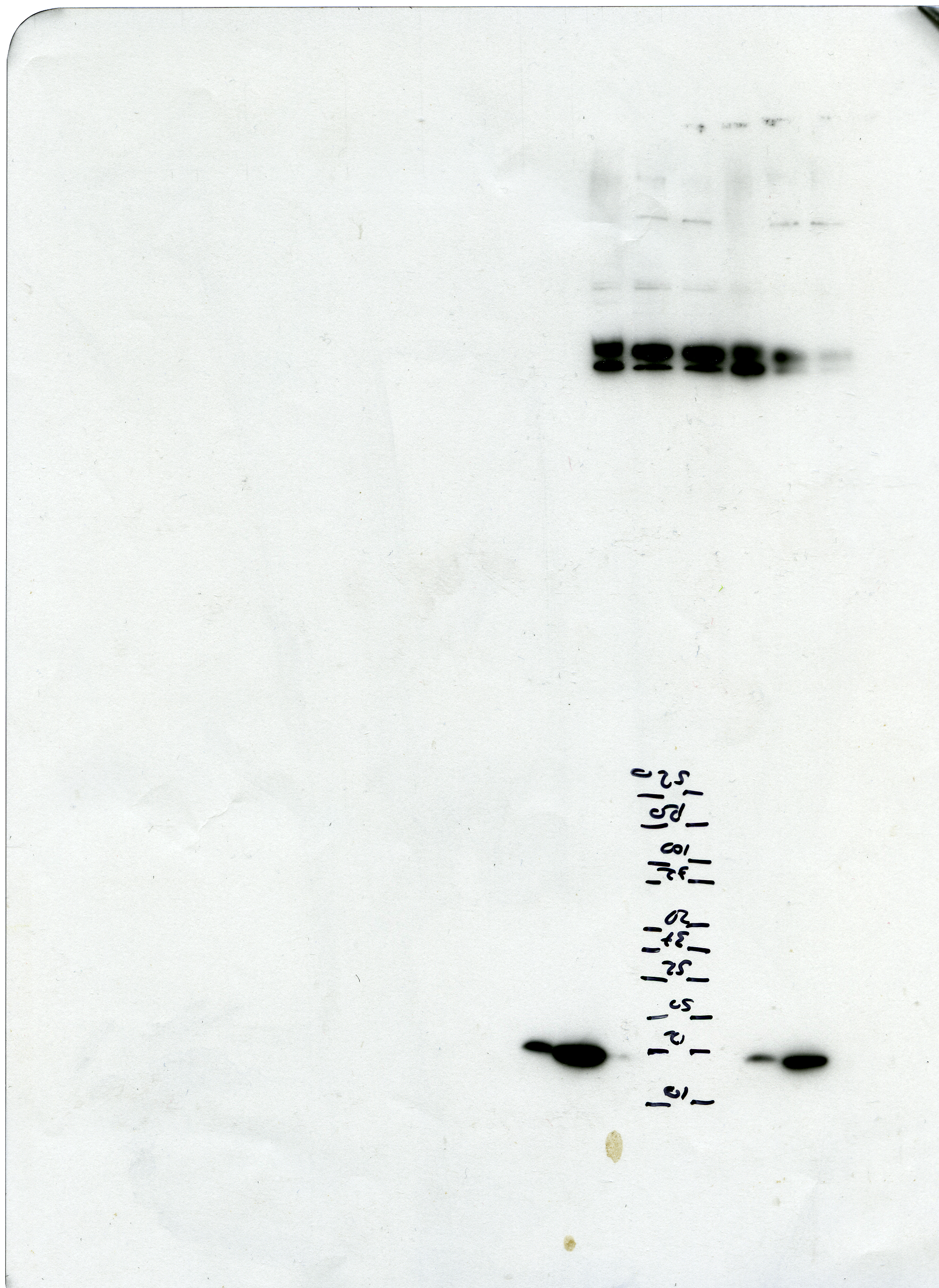

Supplement: Figure 6—source data 2. [file elife-84034-fig6-data2.zip › Figure 6 - source data 2.tif]

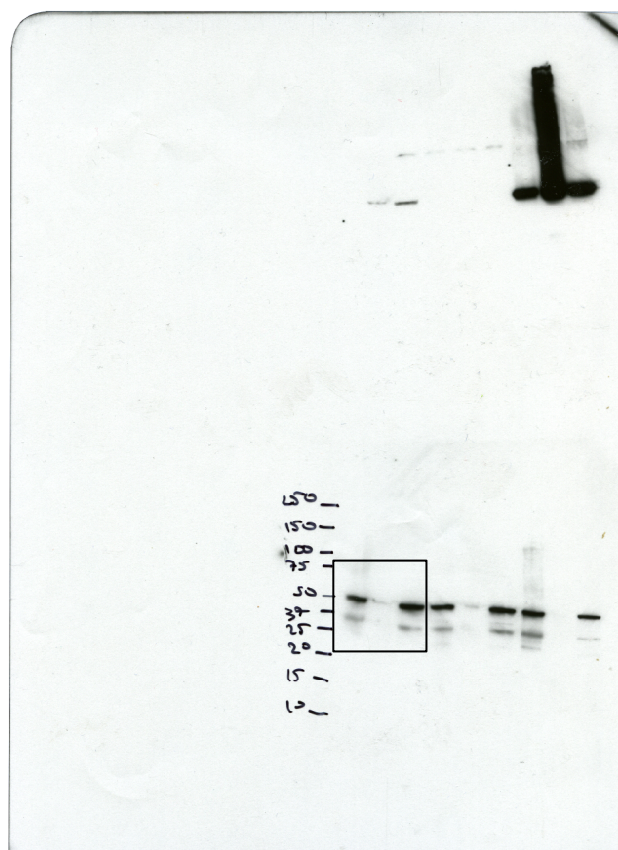

Supplement: Figure 6—source data 3. [file elife-84034-fig6-data3.zip › Figure 6 - source data 3.pdf]

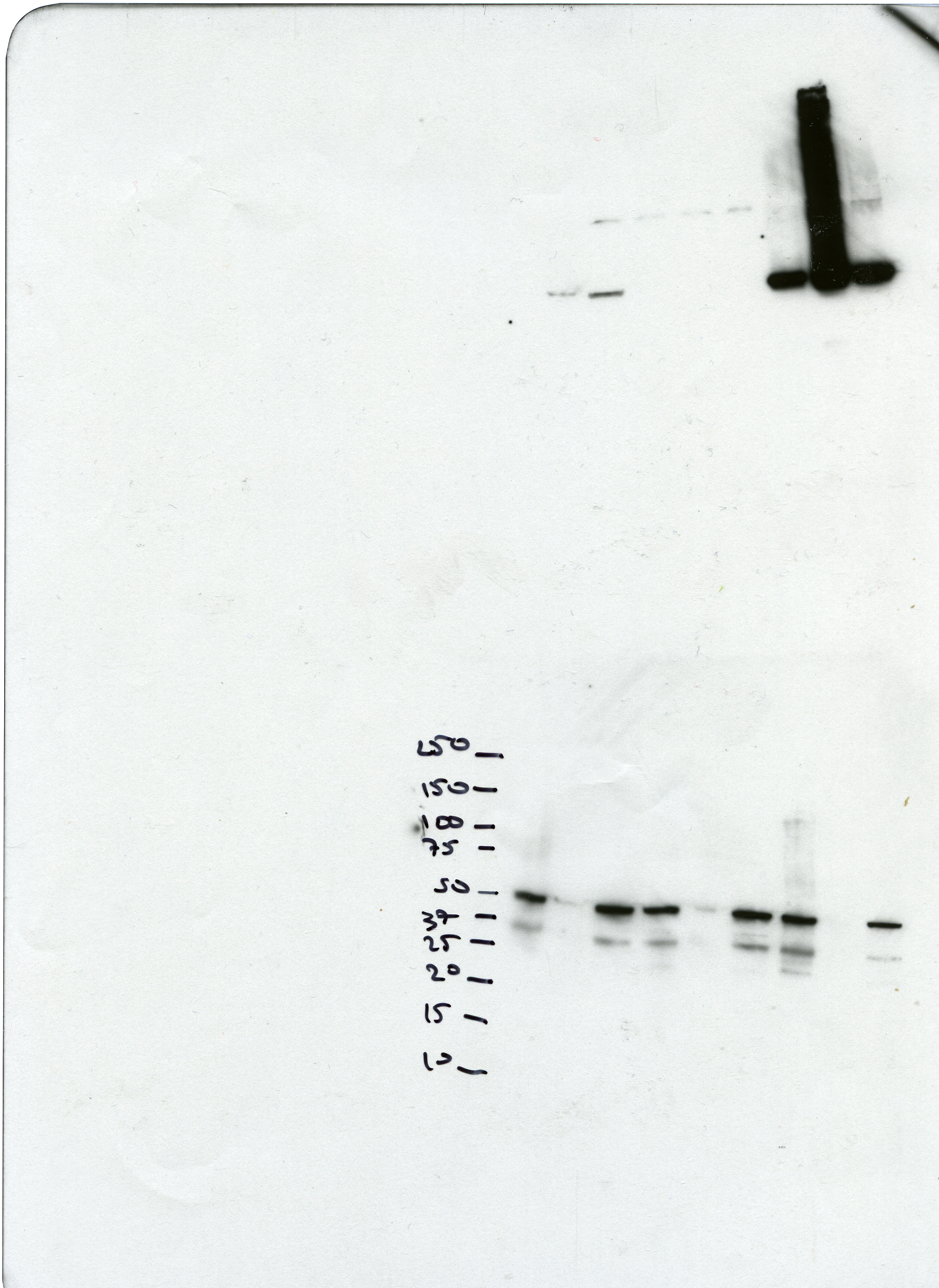

Supplement: Figure 6—source data 3. [file elife-84034-fig6-data3.zip › Figure 6 - source data 3.tif]

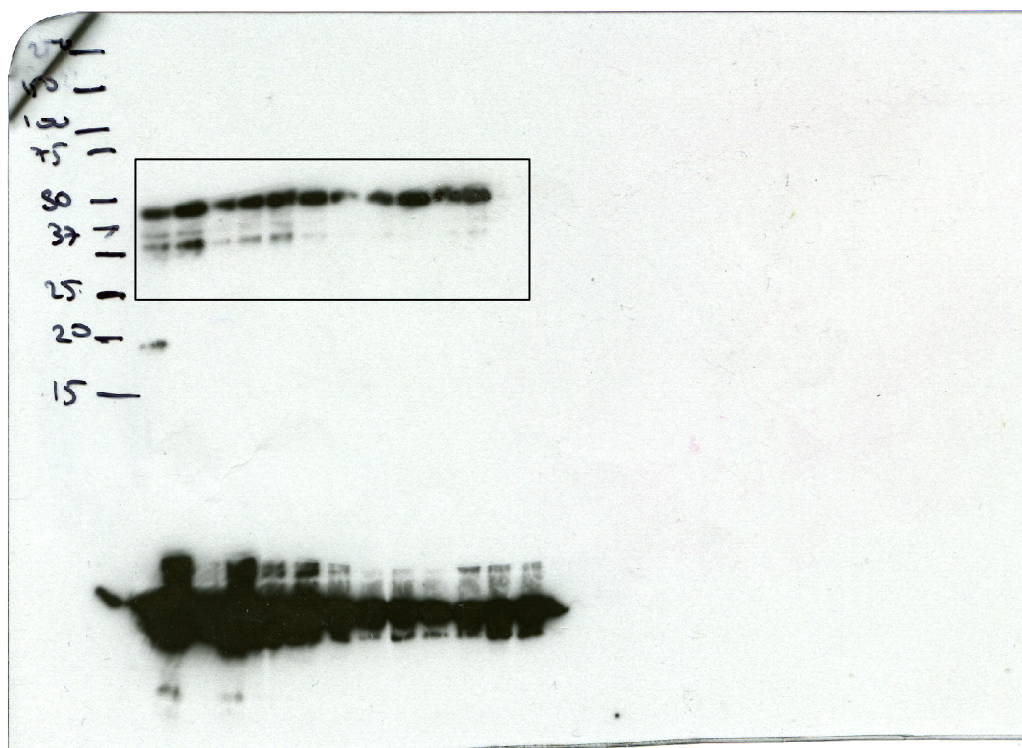

Supplement: Figure 6—source data 4. [file elife-84034-fig6-data4.zip › Figure 6 - source data 4.pdf]

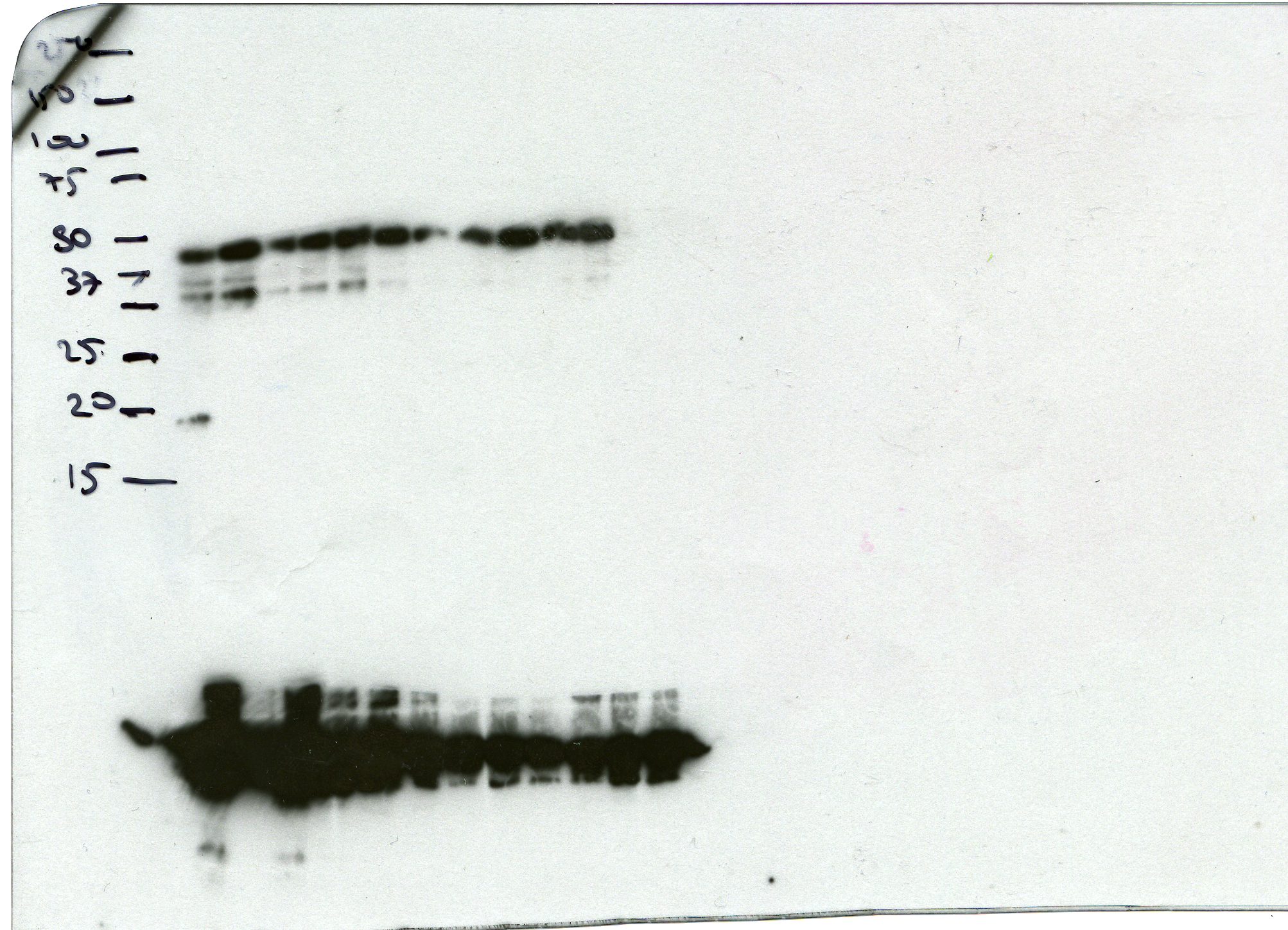

Supplement: Figure 6—source data 4. [file elife-84034-fig6-data4.zip › Figure 6 - source data 4.tif]

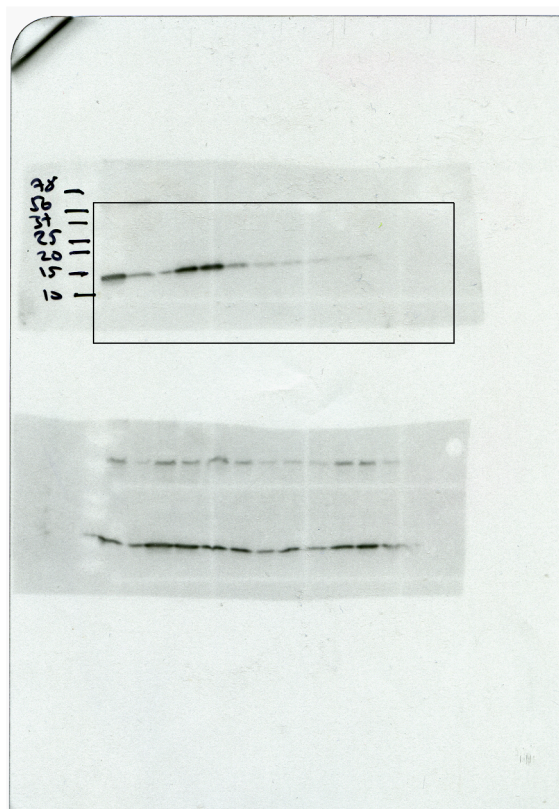

Supplement: Figure 6—source data 5. [file elife-84034-fig6-data5.zip › Figure 6 - source data 5.pdf]

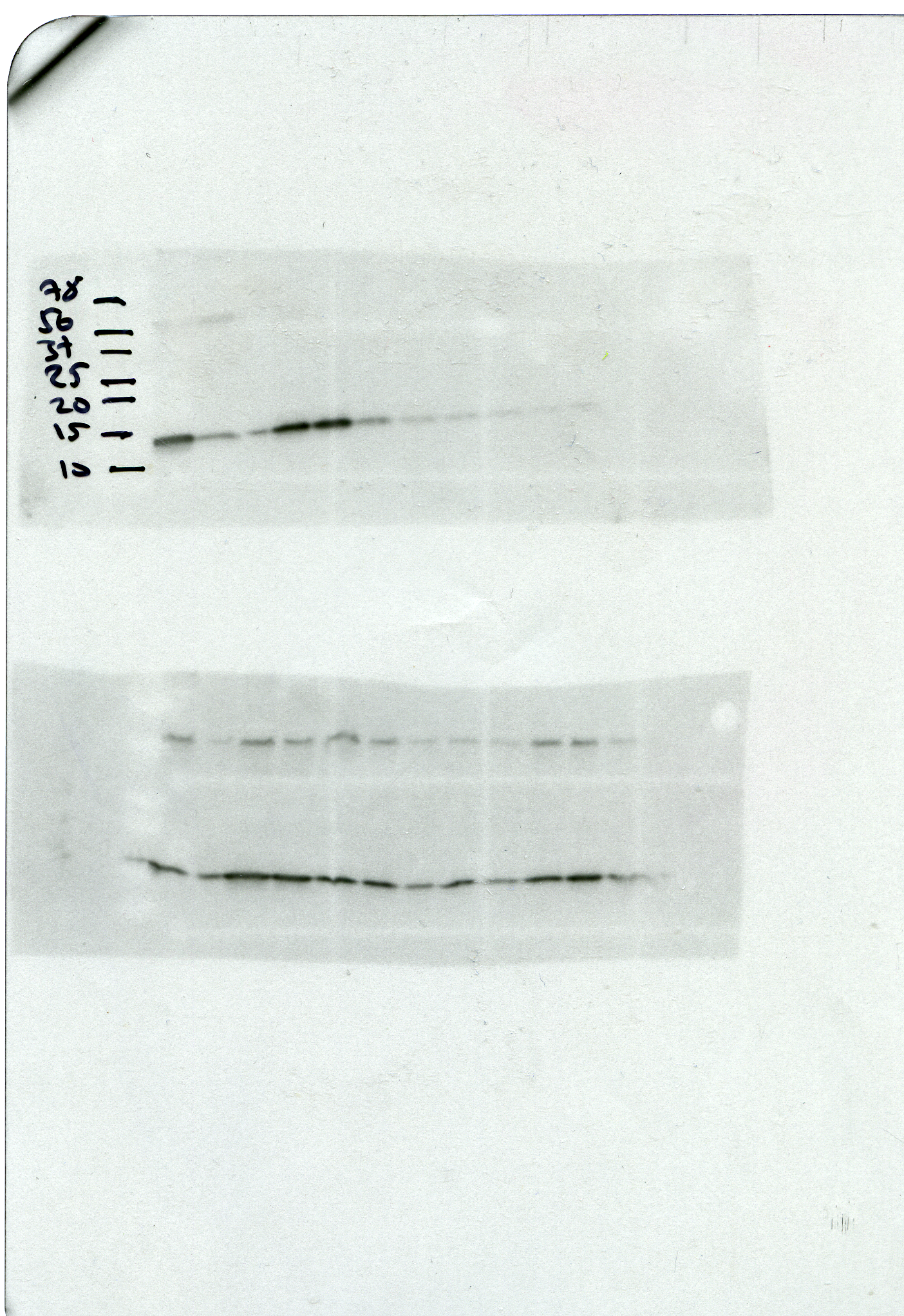

Supplement: Figure 6—source data 5. [file elife-84034-fig6-data5.zip › Figure 6 - source data 5.tif]

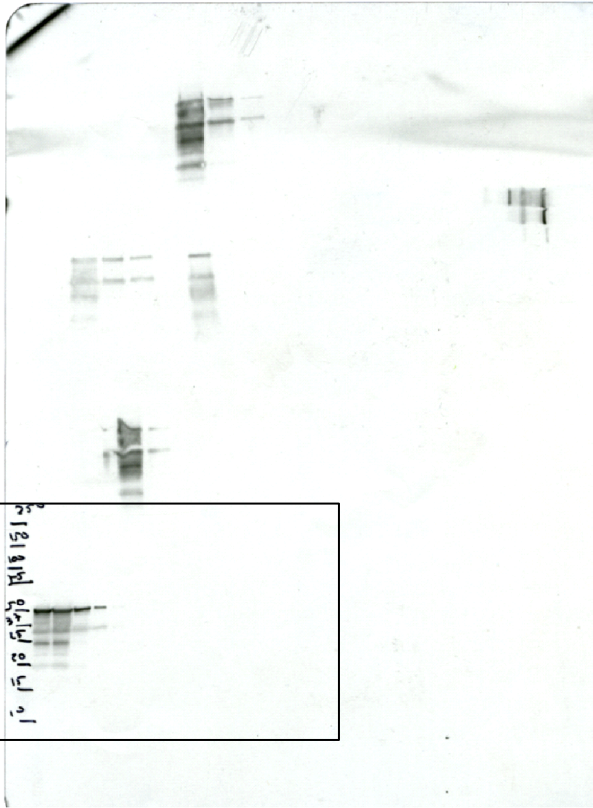

Supplement: Figure 6—source data 6. [file elife-84034-fig6-data6.zip › Figure 6 - source data 6.pdf]

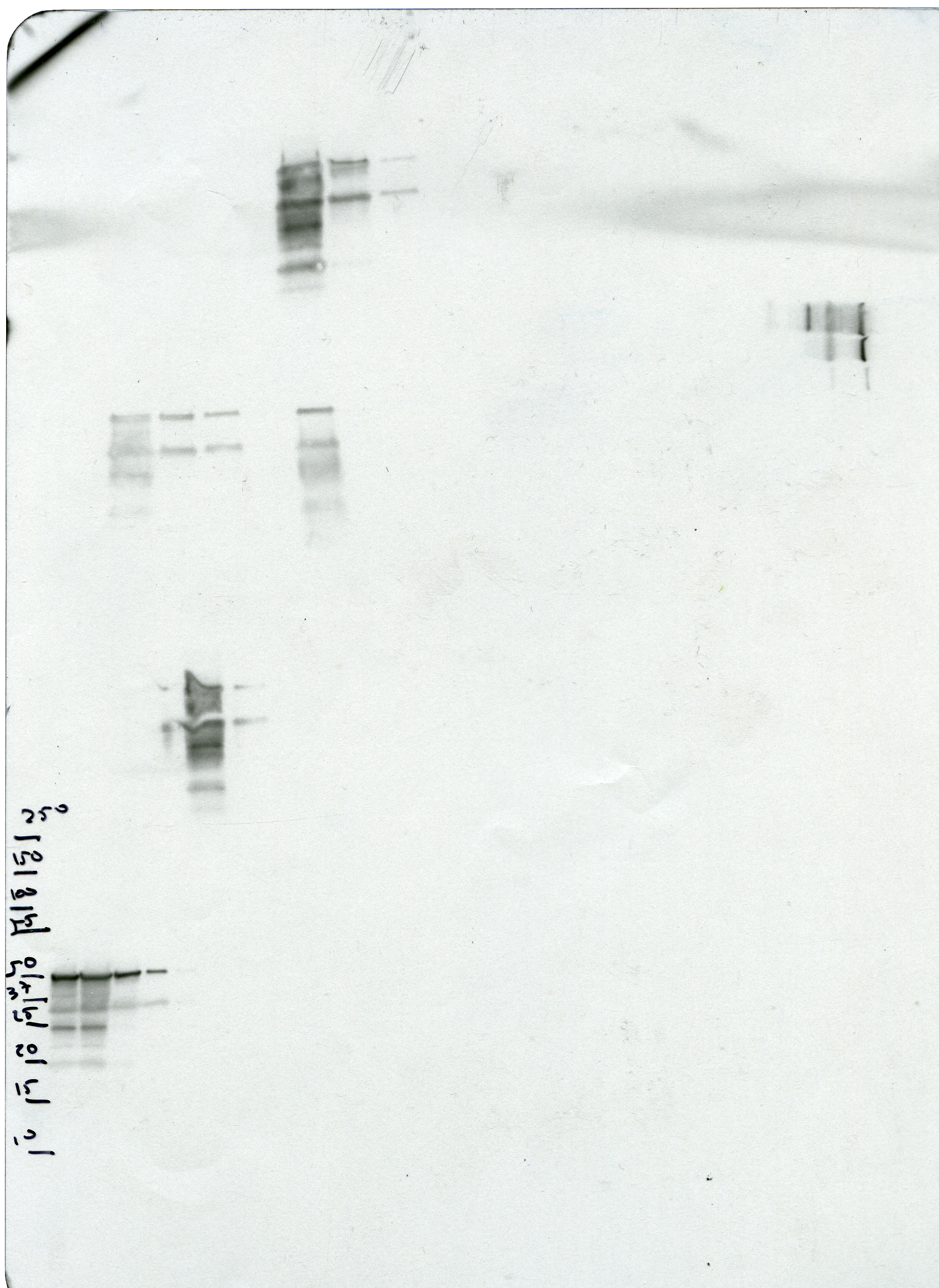

Supplement: Figure 6—source data 6. [file elife-84034-fig6-data6.zip › Figure 6 - source data 6.tif]

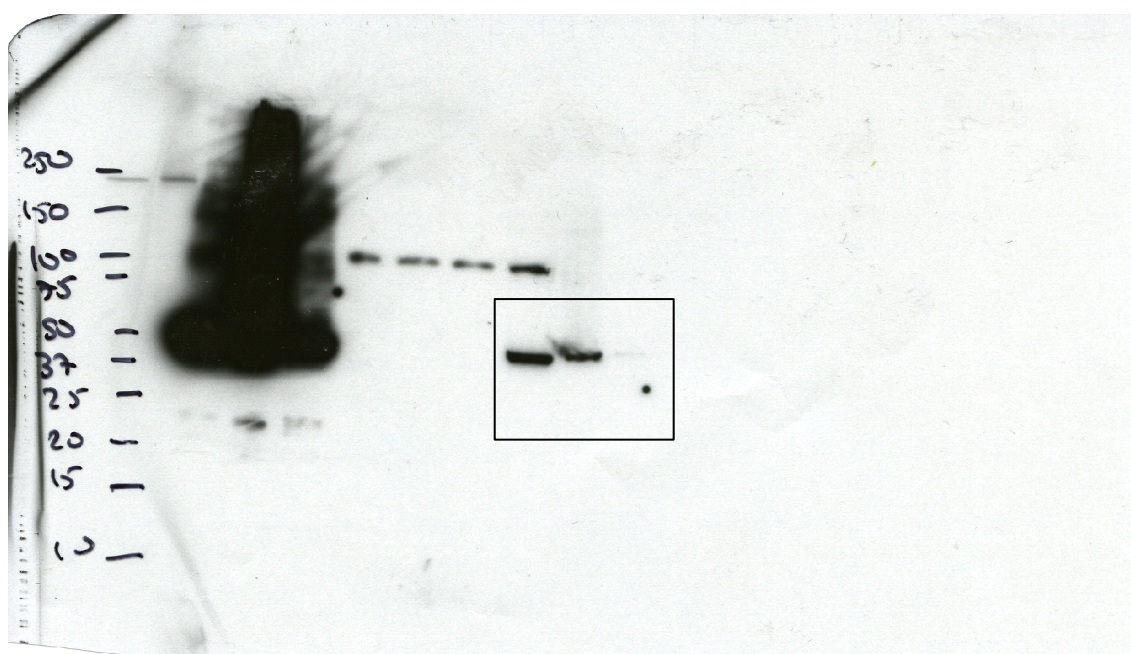

Supplement: Figure 6—figure supplement 1—source data 1. [file elife-84034-fig6-figsupp1-data1.zip › Figure 6 - figure supplement 1 - source data 1.pdf]

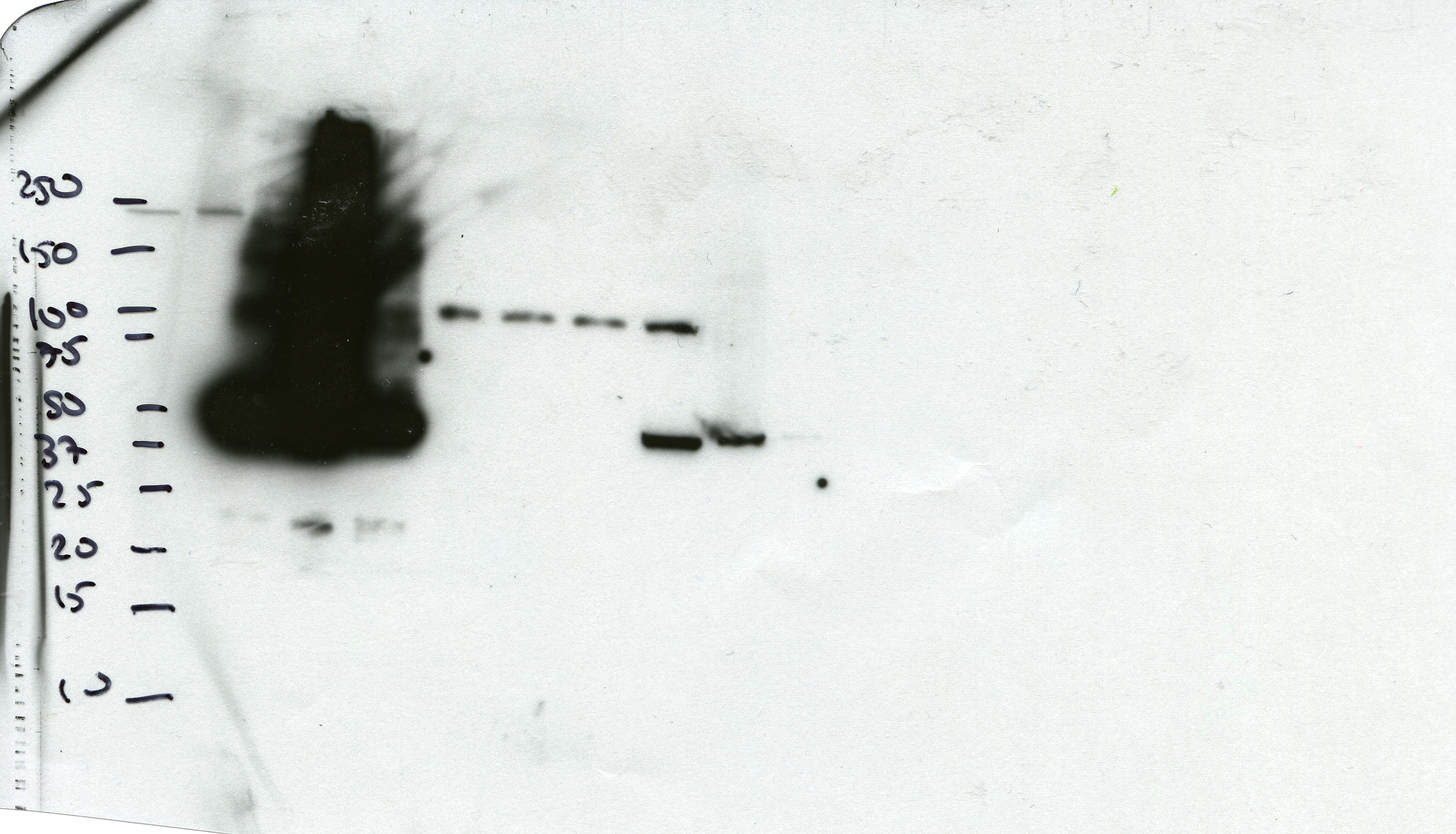

Supplement: Figure 6—figure supplement 1—source data 1. [file elife-84034-fig6-figsupp1-data1.zip › Figure 6 - figure supplement 1 - source data 1.tif]

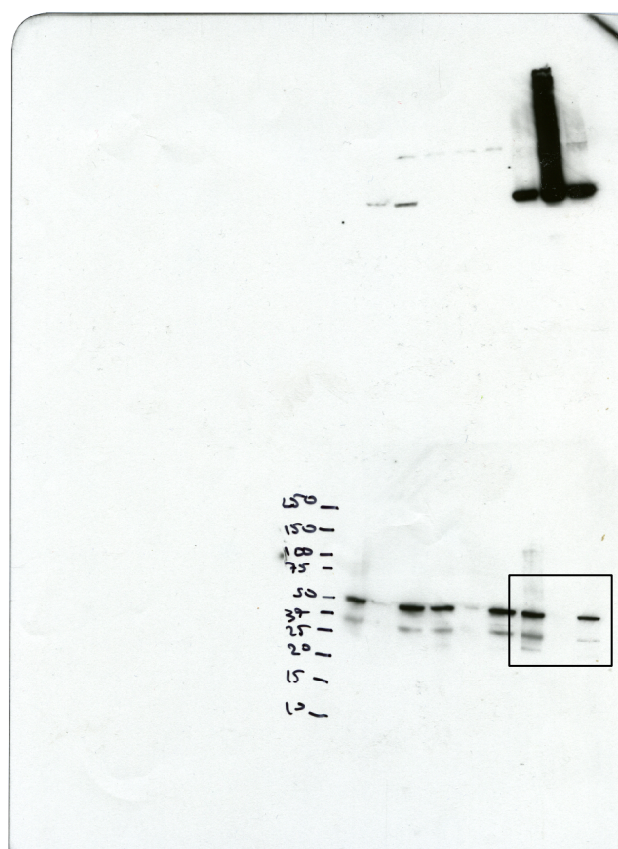

Supplement: Figure 6—figure supplement 1—source data 3. [file elife-84034-fig6-figsupp1-data3.zip › Figure 6 - figure supplement 1 - source data 3.pdf]

Srt1  
(green)  
Hxk1  
(red)

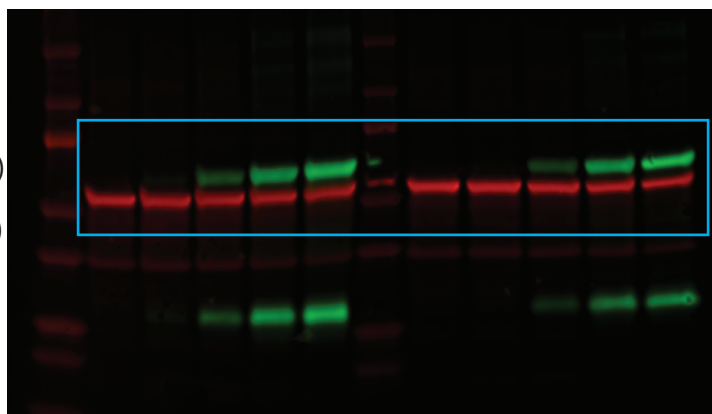

Supplement: Figure 6—figure supplement 1—source data 4. [file elife-84034-fig6-figsupp1-data4.zip › Figure 6 - figure supplement 1 - source data 4.pdf]

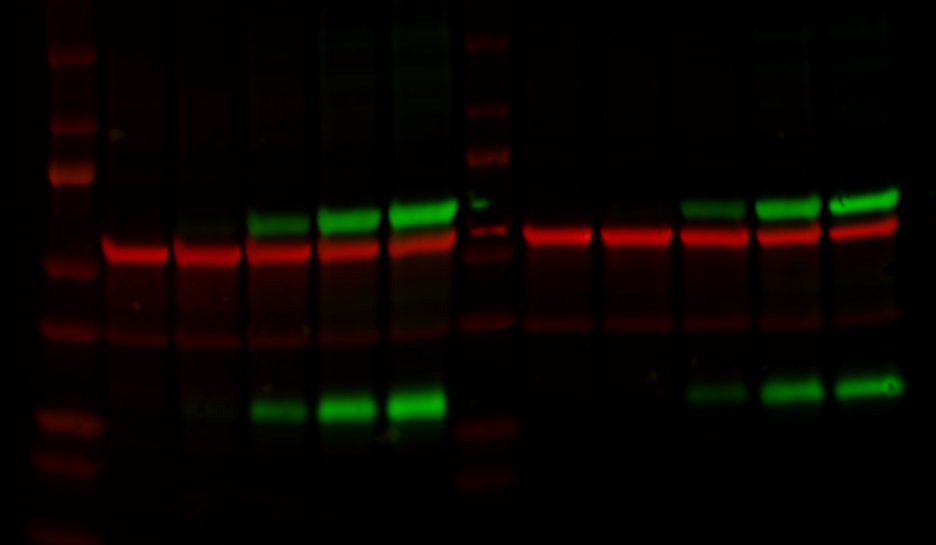

Supplement: Figure 6—figure supplement 1—source data 4. [file elife-84034-fig6-figsupp1-data4.zip › Figure 6 - figure supplement 1 - source data 4.tif]

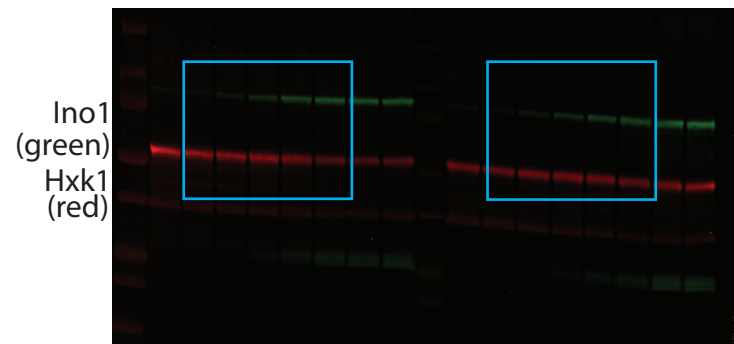

Supplement: Figure 6—figure supplement 1—source data 5. [file elife-84034-fig6-figsupp1-data5.zip › Figure 6 - figure supplement 1 - source data 5.pdf]

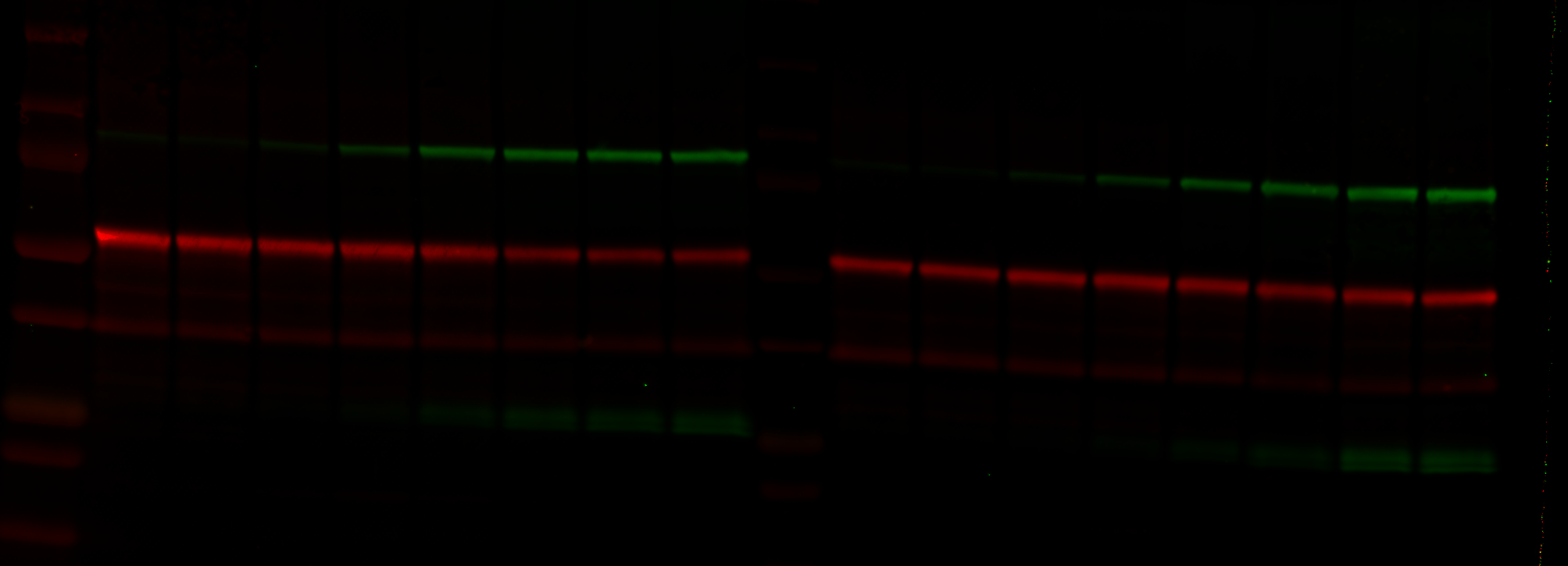

Supplement: Figure 6—figure supplement 1—source data 5. [file elife-84034-fig6-figsupp1-data5.zip › Figure 6 - figure supplement 1 - source data 5.tif]

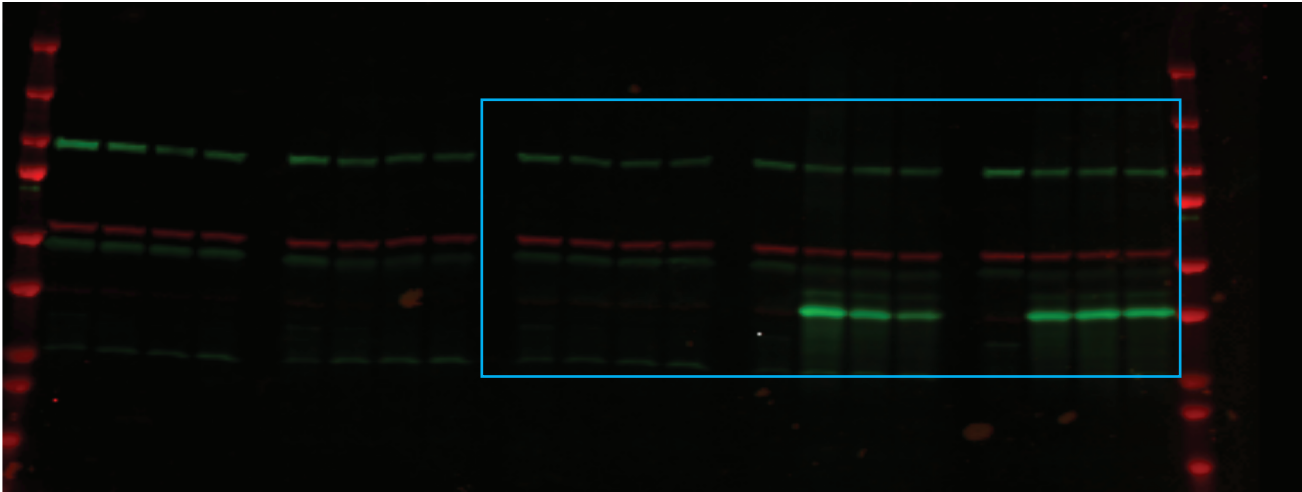

Gut2  
Hxk1  
Pho92

Supplement: Figure 6—figure supplement 1—source data 6. [file elife-84034-fig6-figsupp1-data6.zip › Figure 6 - figure supplement 1 - source data 6.pdf]

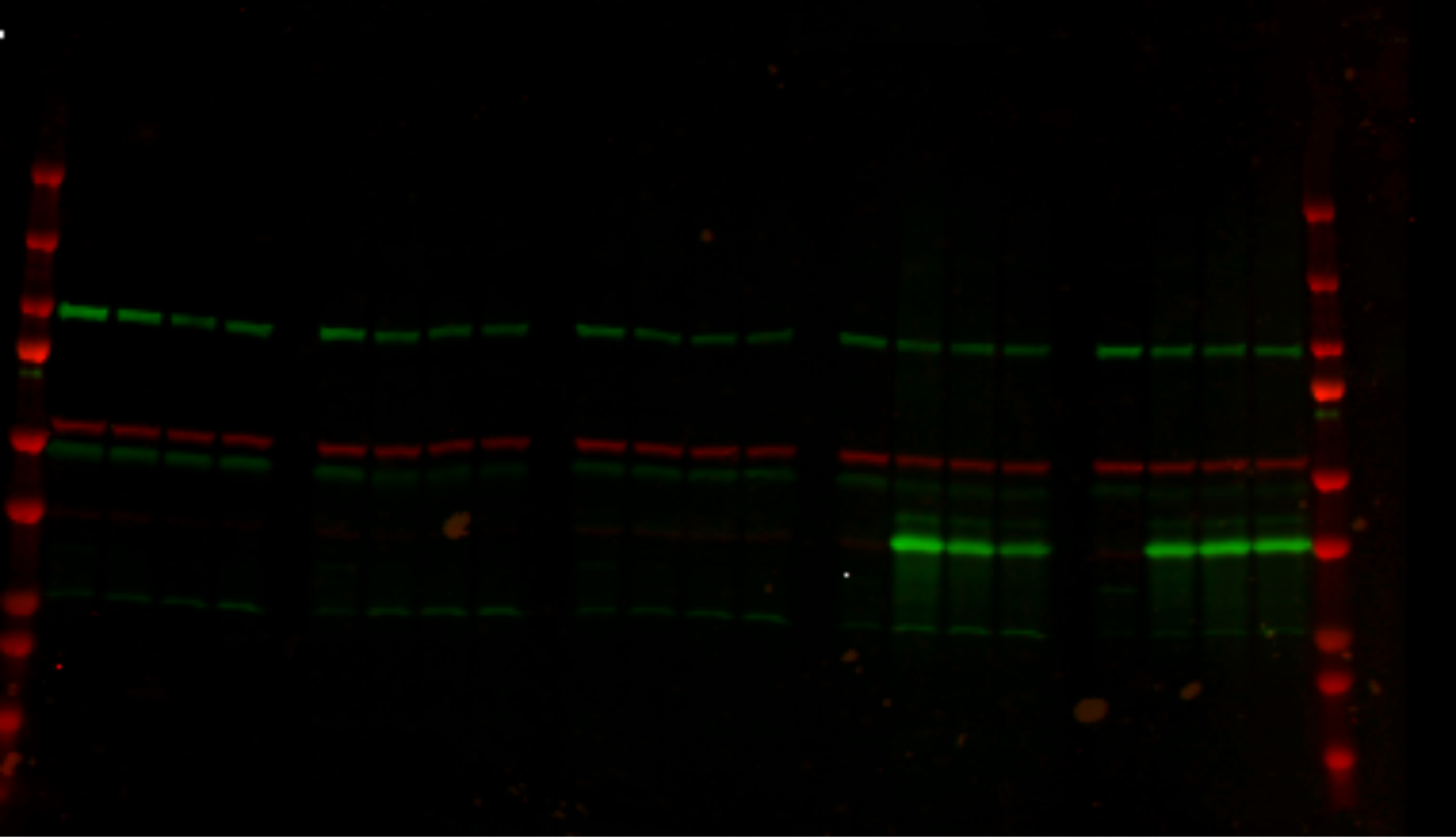

Supplement: Figure 6—figure supplement 1—source data 6. [file elife-84034-fig6-figsupp1-data6.zip › Figure 6 - figure supplement 1 - source data 6.tif]

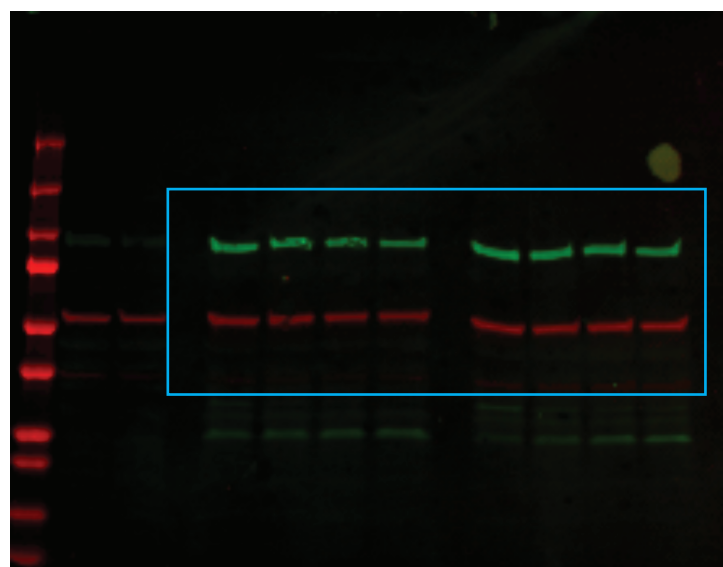

Gut2

Hxk1

Supplement: Figure 6—figure supplement 1—source data 7. [file elife-84034-fig6-figsupp1-data7.zip › Figure 6 - figure supplement 1 - source data 7.pdf]

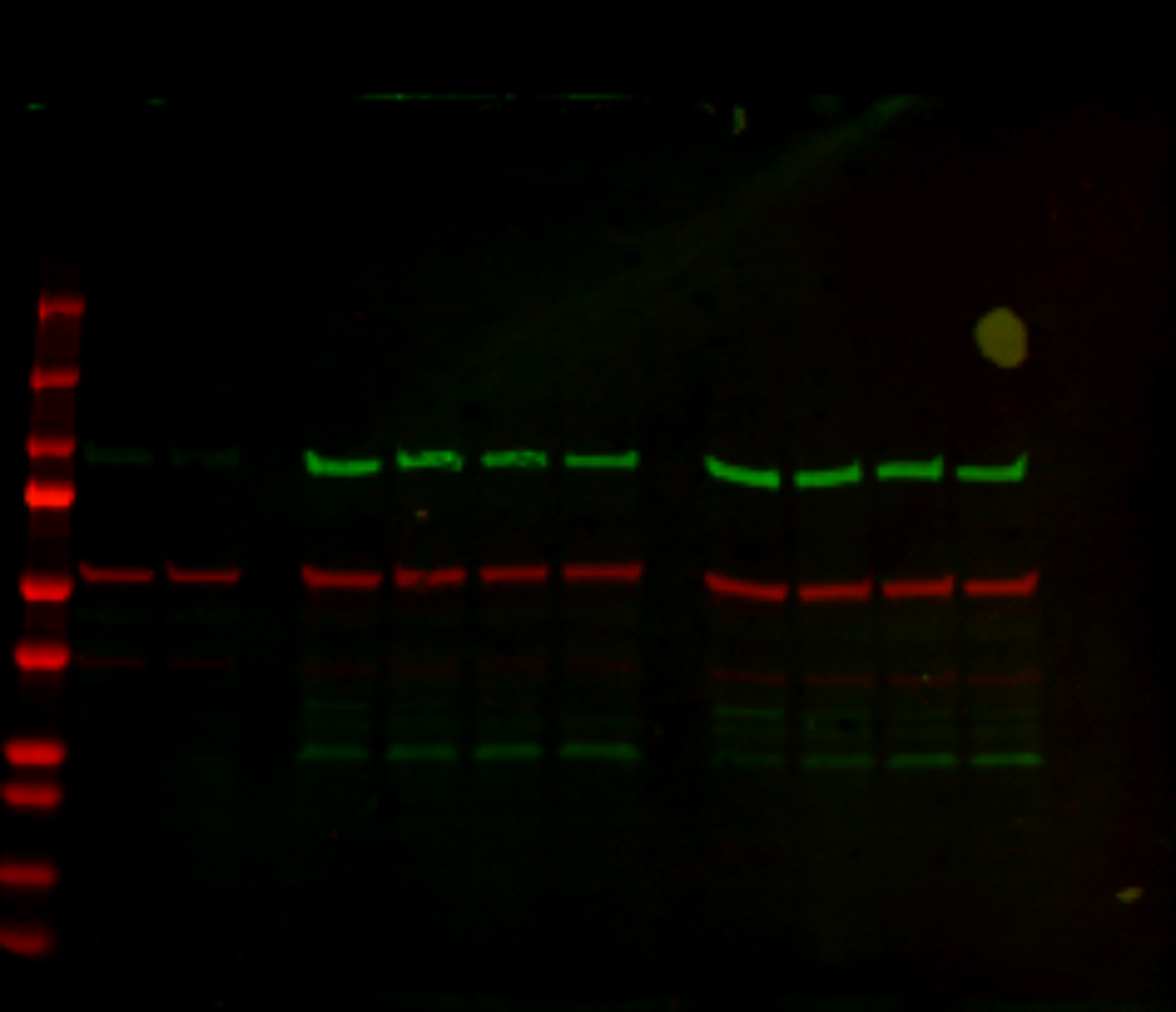

Supplement: Figure 6—figure supplement 1—source data 7. [file elife-84034-fig6-figsupp1-data7.zip › Figure 6 - figure supplement 1 - source data 7.tif]

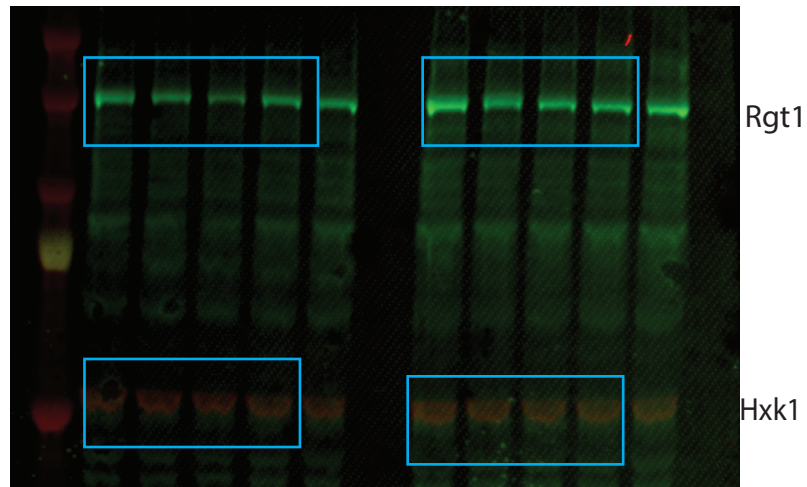

Supplement: Figure 6—figure supplement 1—source data 8. [file elife-84034-fig6-figsupp1-data8.zip › Figure 6 - figure supplement 1 - source data 8.pdf]

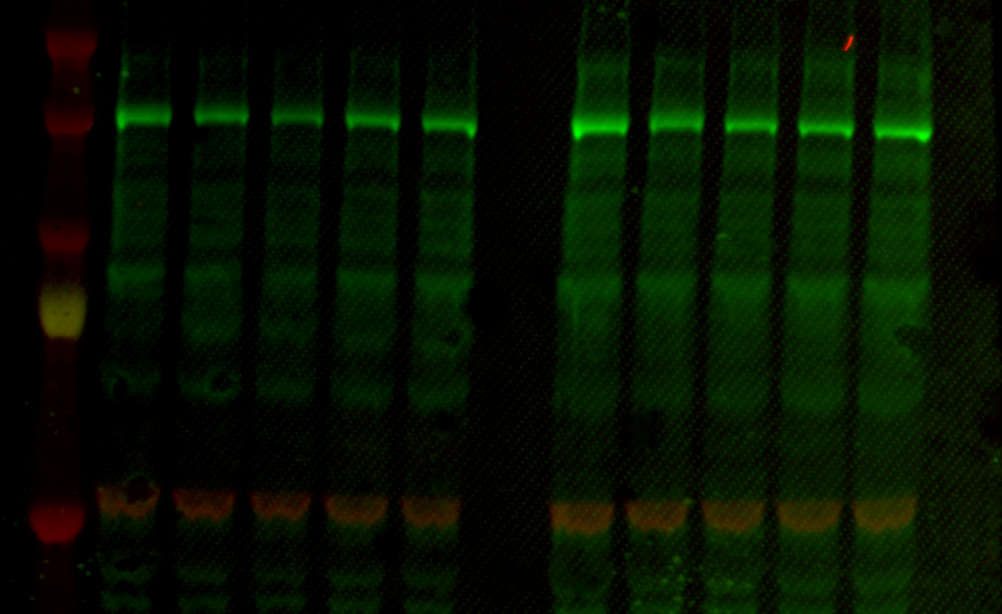

Supplement: Figure 6—figure supplement 1—source data 8. [file elife-84034-fig6-figsupp1-data8.zip › Figure 6 - figure supplement 1 - source data 8.tif]

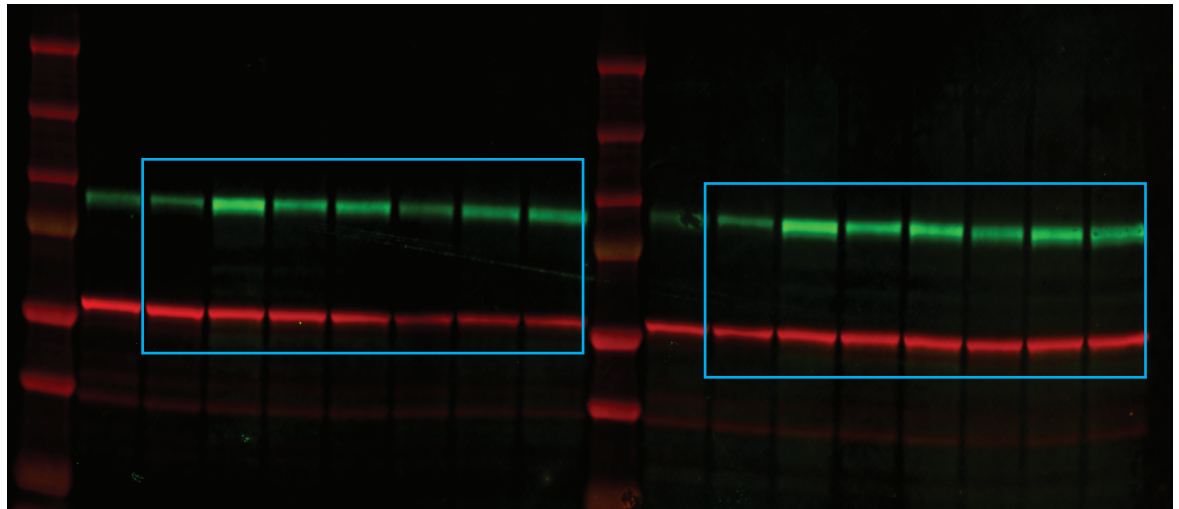

Bdf2

Hxk1

Supplement: Figure 6—figure supplement 1—source data 9. [file elife-84034-fig6-figsupp1-data9.zip › Figure 6 - figure supplement 1 - source data 9.pdf]

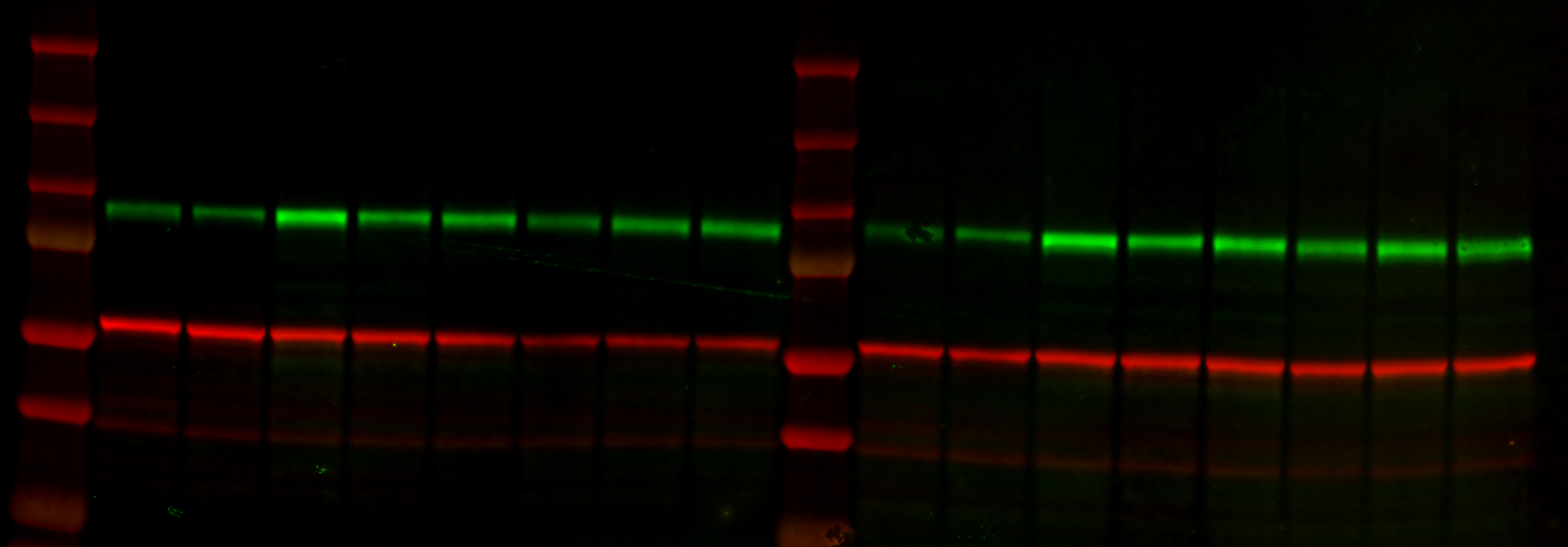

Supplement: Figure 6—figure supplement 1—source data 9. [file elife-84034-fig6-figsupp1-data9.zip › Figure 6 - figure supplement 1 - source data 9.tif]
