# Supplementary material for: N6-methyladenosine (m6A) reader Pho92 is recruited co-transcriptionally and couples translation to mRNA decay to promote meiotic fitness in yeast
Source: eLife. 2022 Nov 24;11:e84034. doi: 10.7554/eLife.84034 (PMC9731578; doi:10.7554/eLife.84034)

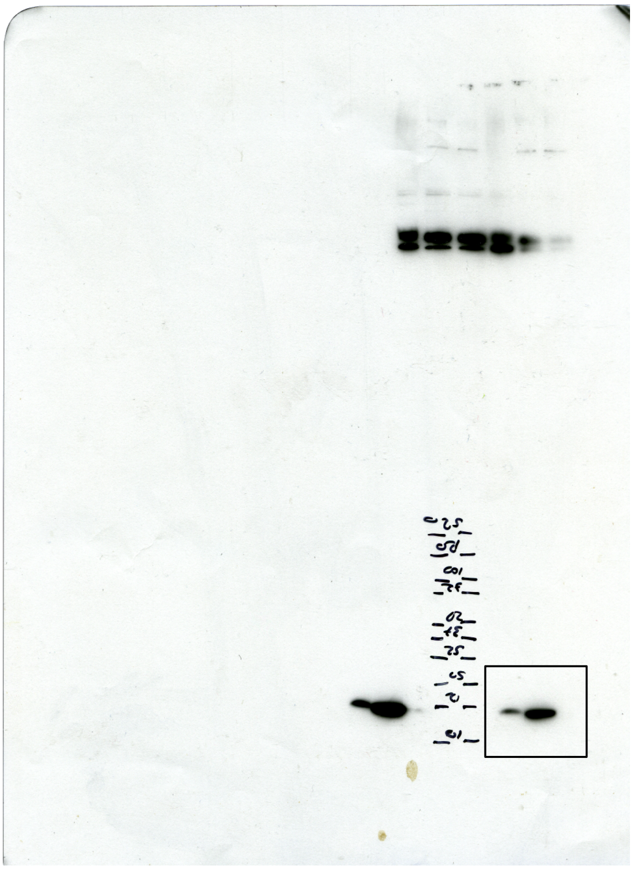

Figure 6 – figure supplement 1E

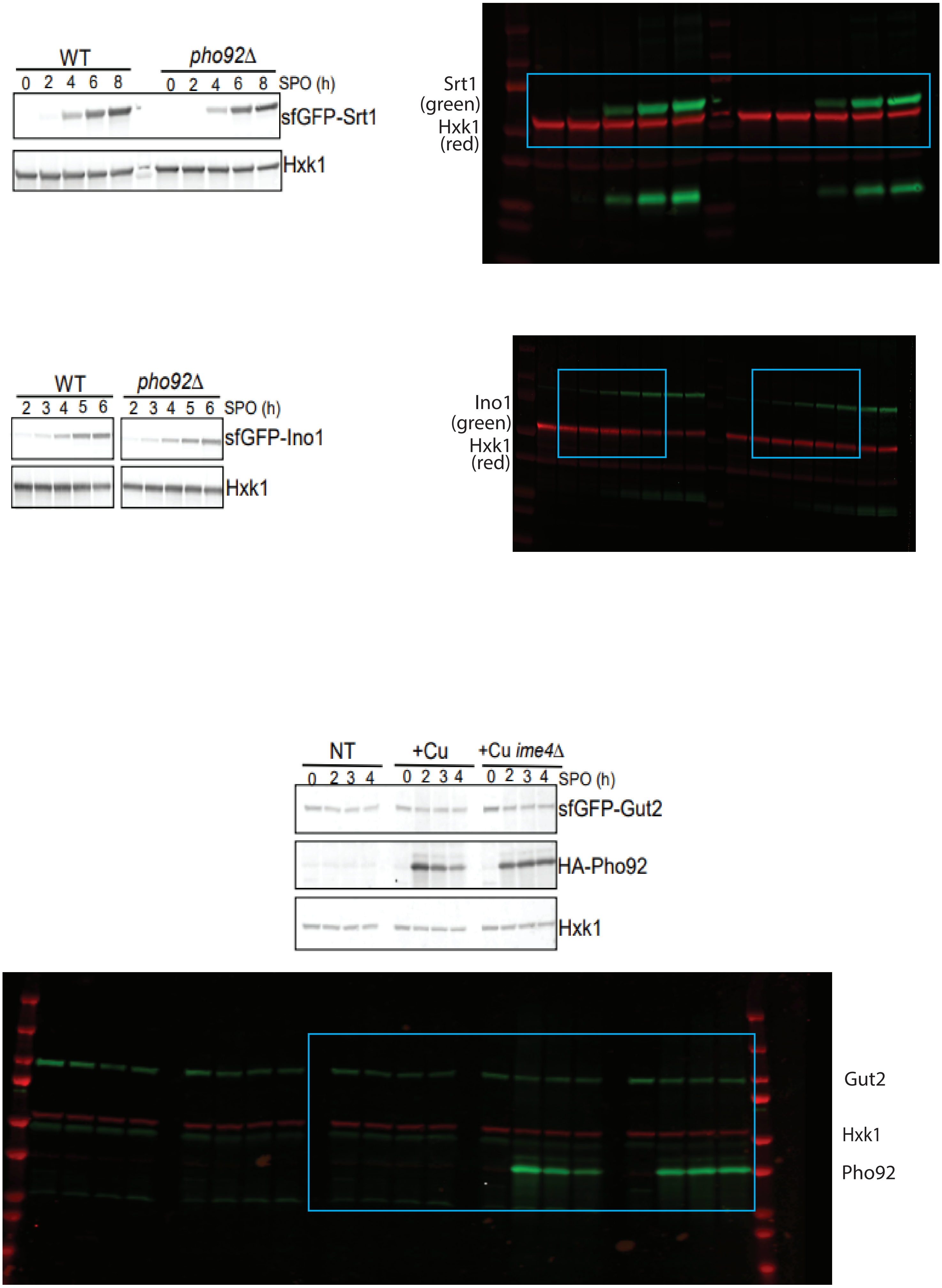

Figure 6 – figure supplement 1F

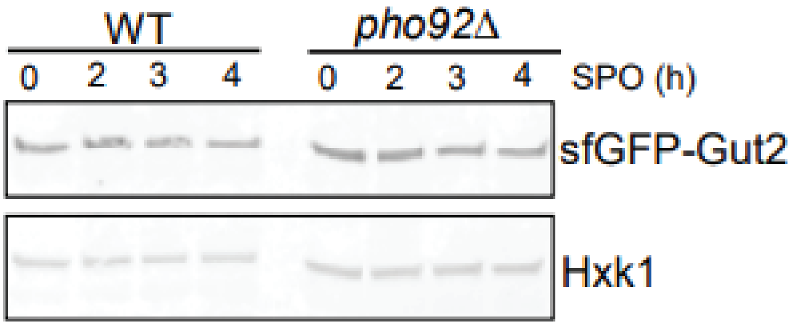

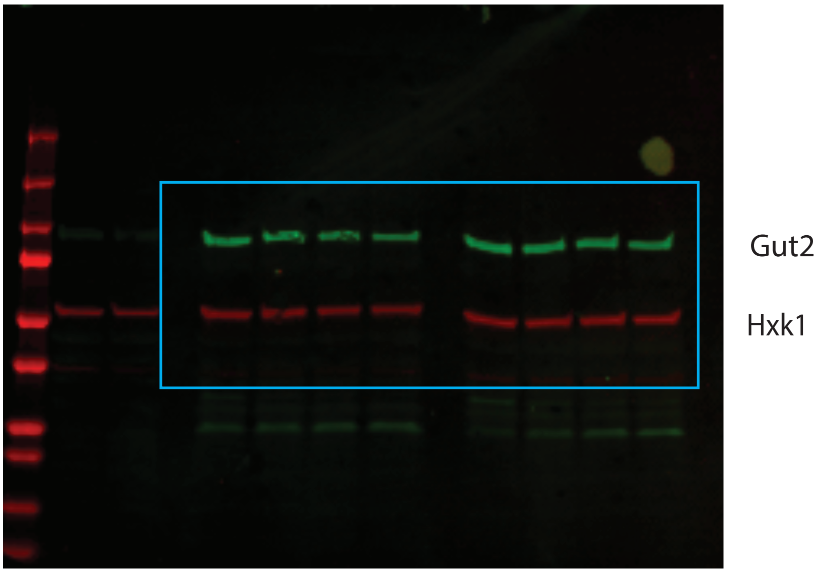

Figure 6 – figure supplement 1G

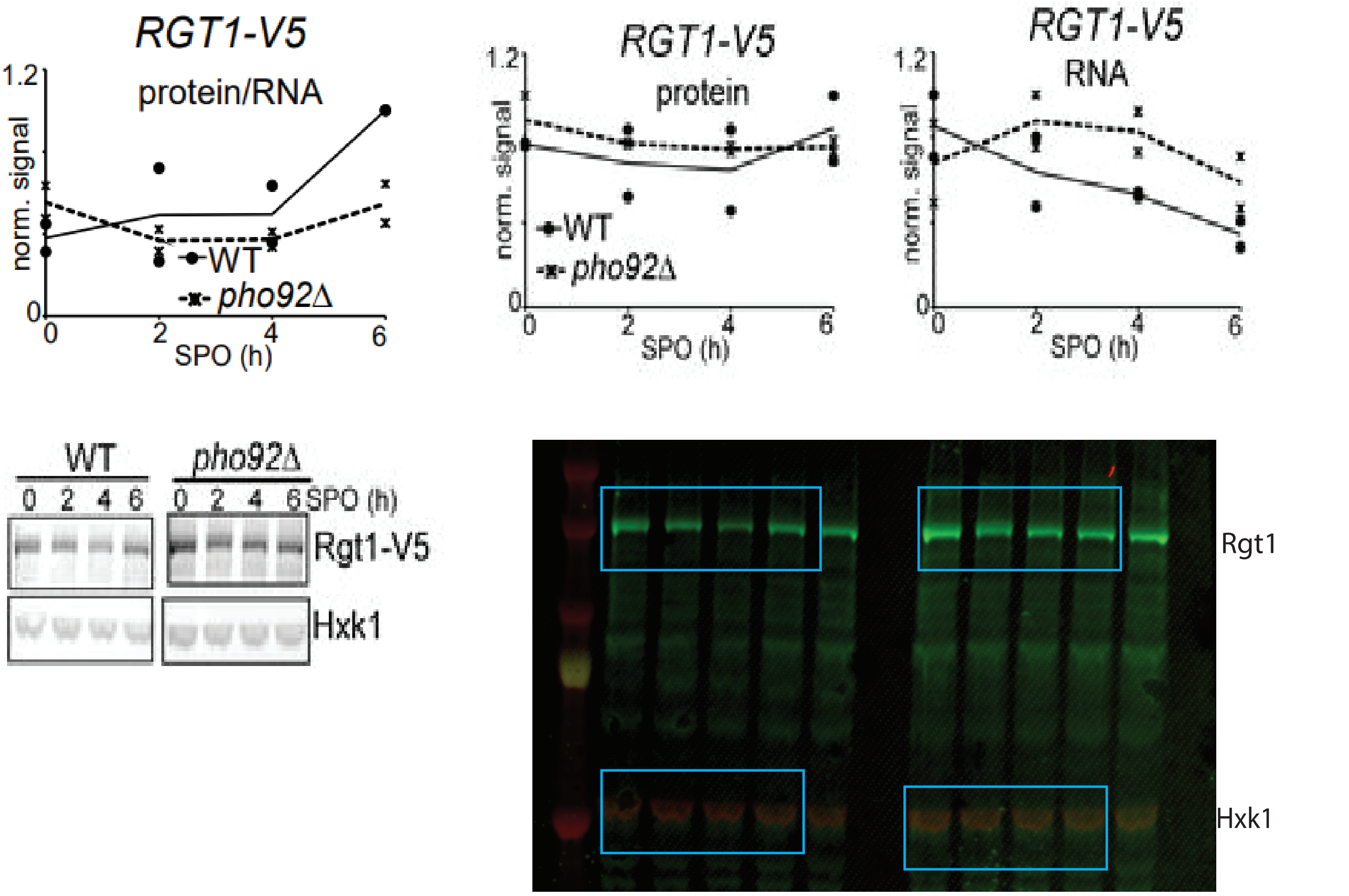

Figure 6 – figure supplement 1H

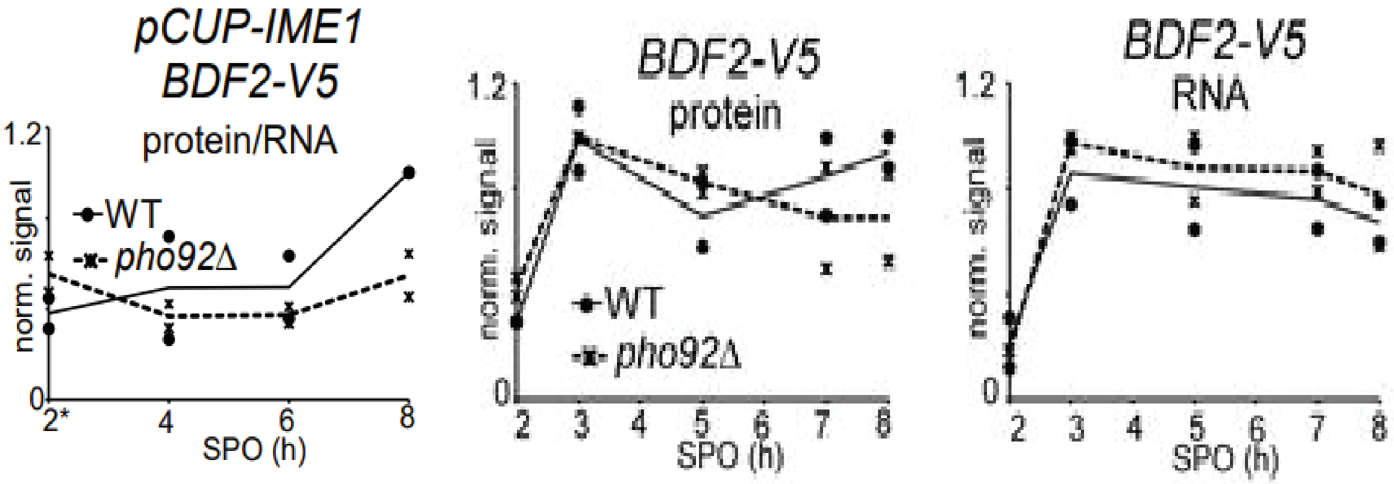

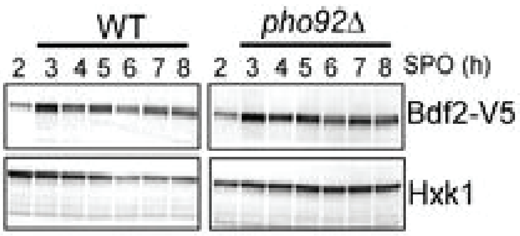

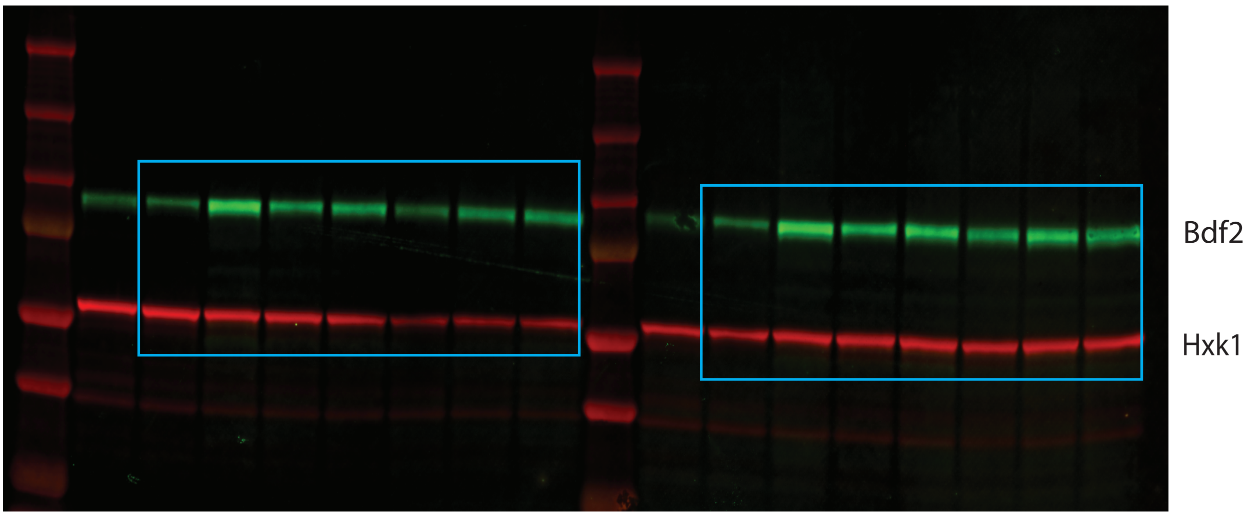

Supplement: Figure 6—figure supplement 1—source data 2. [file elife-84034-fig6-figsupp1-data2.zip › Figure 6 - figure supplement 1 - source data 2.pdf]
