# Supplementary material for: N6-methyladenosine (m6A) reader Pho92 is recruited co-transcriptionally and couples translation to mRNA decay to promote meiotic fitness in yeast
Source: eLife. 2022 Nov 24;11:e84034. doi: 10.7554/eLife.84034 (PMC9731578; doi:10.7554/eLife.84034)
Supplement: Supplementary file 2. [file elife-84034-supp2.docx]

**Supplementary file 2. Plasmids used in this study**

| Plasmid No. | Name |
| --- | --- |
| \| FW P768 \| \| --- \| | pGEX-6P-1 |
| FW P769 | pGEX-6P-1-Gis2 |
| FW P759 | pGEX-6P-1-Pho92-FL |
| FW P760 | pGEX-6P-1-Pho92-NTDd |
| FW P761 | pGEX-6P-1-Pho92-YTHd |
| FW P718 | pK3FS-PYK1 (Addgene Plasmid #85777) |
| FW P719 | pK3FS-CYC1 (Addgene Plasmid #85779) |
| FW P762 | p373-Cup1-HA-Pho92-WT |
| FW P763 | p373-Cup1-HA-Pho92-W177A |
| FW P373 | pNH604 |
| FW P782 | Ime4-V5 TRP WT integration plasmid |
| FW P783 | Ime4-V5 TRP catalytic dead integration plasmid |
